# Muscle eosinophilia is a hallmark of chronic disease in facioscapulohumeral muscular dystrophy

**DOI:** 10.1093/hmg/ddae019

**Published:** 2024-02-10

**Authors:** Andreia M Nunes, Monique M Ramirez, Enrique Garcia-Collazo, Takako Iida Jones, Peter L Jones

**Affiliations:** Department of Pharmacology, University of Nevada, Reno School of Medicine, 1664 N. Virginia St., Reno, NV 89557, United States; Department of Pharmacology, University of Nevada, Reno School of Medicine, 1664 N. Virginia St., Reno, NV 89557, United States; Department of Pharmacology, University of Nevada, Reno School of Medicine, 1664 N. Virginia St., Reno, NV 89557, United States; Department of Pharmacology, University of Nevada, Reno School of Medicine, 1664 N. Virginia St., Reno, NV 89557, United States; Department of Pharmacology, University of Nevada, Reno School of Medicine, 1664 N. Virginia St., Reno, NV 89557, United States

**Keywords:** FSHD, immune cell, mouse model, human disease

## Abstract

Facioscapulohumeral muscular dystrophy (FSHD) is a progressive myopathy caused by the aberrant increased expression of the *DUX4* retrogene in skeletal muscle cells. The *DUX4* gene encodes a transcription factor that functions in zygotic genome activation and then is silenced in most adult somatic tissues. DUX4 expression in FSHD disrupts normal muscle cell function; however, the downstream pathogenic mechanisms are still unclear. Histologically, FSHD affected muscles show a characteristic dystrophic phenotype that is often accompanied by a pronounced immune cell infiltration, but the role of the immune system in FSHD is not understood. Previously, we used *ACTA1;FLExDUX4* FSHD-like mouse models varying in severity as discovery tools to identify increased Interleukin 6 and microRNA-206 levels as serum biomarkers for FSHD disease severity. In this study, we use the *ACTA1;FLExDUX4* chronic FSHD-like mouse model to provide insight into the immune response to DUX4 expression in skeletal muscles. We demonstrate that these FSHD-like muscles are enriched with the chemoattractant eotaxin and the cytotoxic eosinophil peroxidase, and exhibit muscle eosinophilia. We further identified muscle fibers with positive staining for eosinophil peroxidase in human FSHD muscle. Our data supports that skeletal muscle eosinophilia is a hallmark of FSHD pathology.

## Introduction

Facioscapulohumeral muscular dystrophy (FSHD) is a progressive myopathy that affects women and men of all ages with an estimated worldwide prevalence of ~1 in 8300–15 000 [1–3]. FSHD is caused by aberrant expression of the Double homeobox 4 (*DUX4*) retrogene in skeletal muscle as a consequence of epigenetic dysregulation of the 4q35 D4Z4 macrosatellite repeat array [4–8]. *DUX4* encodes a transcription factor that is expressed at the 4-cell stage and plays an important role in the early stages of human embryonic development before becoming epigenetically silenced [9, 10]. However, failure to silence *DUX4* in FSHD leads to aberrant expression in skeletal muscle cells and initiates a cascade of events that ultimately lead to pathology [4, 7, 11, 12].

The presence of immune cell infiltrates in FSHD patient muscle is well documented [13–17]. Early observations from FSHD muscle biopsies reported a moderate correlation between mononuclear cell infiltration in the muscle and increased levels of serum creatine kinase [15]. Later studies confirmed the presence of both B cells and T cells [18], with endomysial infiltrates showing an abundance of CD8^+^ cells and perivascular infiltrates mainly composed of CD4^+^ cells [14]. This inflammatory cell profile is accompanied by an increase in the serum levels of cytokines/chemokines such as CCL5/RANTES, TNF-α, IFN-α and IL-6 [19, 20]. More importantly, the serum levels of IL-6 moderately correlate with disease severity [20]. Other studies have further supported the immune signature in FSHD by reporting the expression of immune-related genes in DUX4-fl transduced primary myoblasts [21] and human biopsies [22]. Insights into immune-related disease mechanisms include the identification of membrane attack complex (MAC) deposits in non-necrotic fibers of FSHD patient biopsies and an increase in the complement system-associated signaling pathway in muscle and plasma specimens from FSHD patients [23, 24]. In addition, studies documenting the expression of the human leukocyte antigen (HLA) ABC in muscle fibers from patient biopsies have supported the hypothesis that FSHD muscles present auto-antigenic peptides [13, 14]. However, a recent study by Greco *et al*. revealed that FSHD patient and healthy control sera display equivalent reactivity to muscle antigens, arguing against this long-standing hypothesis [25].

Overall, the immune response profile and impact in FSHD pathology remain largely unknown. To study DUX4-mediated disease mechanisms in vivo, our laboratory recently developed an FSHD-like mouse model, the *ACTA1-MCM; FLExDUX4* mouse*,* that exhibits mosaic DUX4 expression in skeletal muscle similar to the pattern found in FSHD patients [26, 27]. This bi-transgenic mouse model is generated by crossing the *FLExDUX4* mouse, which contains an inverted and floxed human *DUX4* gene targeted to the *Rosa26* locus, with the ACTA1-MerCreMer mouse strain that has expression of a mutated estrogen receptor (Mer) dimer fused with cre (MCM) under the transcriptional control of the *ACTA1* (human skeletal actin) promoter [28]. The resulting bi-transgenic mice undergo transgene recombination to the sense orientation in the presence of nuclear MCM, resulting in mosaic expression of DUX4 in skeletal muscles that leads to FSHD-like pathophysiology. Thus, these mice can be used as a tamoxifen-inducible high DUX4 expression model for testing DUX4-targeted therapeutics. Interestingly, in the absence of tamoxifen induction, cytoplasmic MCM protein will leak into a small fraction of myonuclei, leading to mosaic transgene recombination and expression. This results in a chronic mosaic DUX4 expression model that accumulates muscle pathology as the mice age, thus more accurately recapitulating the FSHD phenotype [26, 27].

In this study, we used our well-established chronic *ACTA1; FLExDUX4* FSHD-like mouse model as a discovery tool to characterize new aspects of the immune response to chronic mosaic expression of DUX4 in skeletal muscle, and confirmed the results in human skeletal muscle samples. We found that FSHD-like expression of DUX4 is associated with muscle eosinophilia and elevated levels of eotaxin and eosinophil peroxidase, which may serve as new muscle biomarkers for FSHD pathology.

## Results

### Skeletal muscles from chronic FSHD-like mice express physiological levels of major histocompatibility complex (MHC) class I

Immune cell infiltration is a well-established hallmark of FSHD muscle pathology [13–17]; however, the role of the immune response in FSHD is still largely unknown. Under physiological conditions, muscle cells express low levels of HLA (human leukocyte antigen) ABC; however, it is highly and widely expressed in muscles from patients with autoimmune idiopathic inflammatory myopathies [29, 30]. Observations of increased HLA ABC on the surface of some myofibers in FSHD biopsies have raised the hypothesis that myofibers from FSHD muscle might present auto-antigenic peptides [13, 14, 31]. However, a recent study provided evidence that the reactivity of FSHD patient sera do not differ from that of healthy controls, casting doubt on the likelihood of an autoantibody-mediated mechanism for FSHD [25].

To better understand the immune responses and immune cell profile in FSHD skeletal muscles, we used the FSHD-like bi-transgenic mouse line *ACTA1-MCM;FLExD*, which, in the absence of tamoxifen induction, is a well-established model of chronic DUX4 expression. The low mosaic expression of DUX4 in this mouse model leads to many aspects of FSHD pathophysiology in skeletal muscle, including increased centrally nucleated myofibers, immune cell infiltration, and reduced muscle regeneration [13, 14, 26, 27, 32–34]. DUX4 expression is detectable as early as 1 month; however, histopathological landmarks are not detected until 3 months of age, with a gradual accumulation of pathology, similar to the situation in FSHD [26].

DUX4 is expressed early in development and in very limited healthy adult tissues, leading to the hypothesis that FSHD may be caused, at least in part, by an autoimmune response to the aberrant skeletal muscle expression of DUX4 and its downstream targets [7] or impaired nonsense-mediated decay [35]. To define the type of immune response to DUX4 expression (i.e. autoimmune vs non-autoimmune), we first analyzed the expression of the murine MHC I, which is homologous to HLA ABC, in the gastrocnemius muscle from chronic FSHD-like mice (Fig. S1). Our results demonstrate that MHC I expression is similar in 6- and 14–18 month-old *ACTA1-MCM/FLExD/+* muscles compared to the respective *ACTA1-MCM/+* age-matched controls (Fig. S1). Thus, muscle with chronic expression of DUX4 does not exhibit the increase in fiber MHC I expression that is typical of autoimmune diseases. These results are consistent with the absence of muscle auto-reactive antibodies in the sera of FSHD patients [25].

### Eotaxin/CCL11 is enriched in the muscles of chronic FSHD-like mice

**Figure 1:**
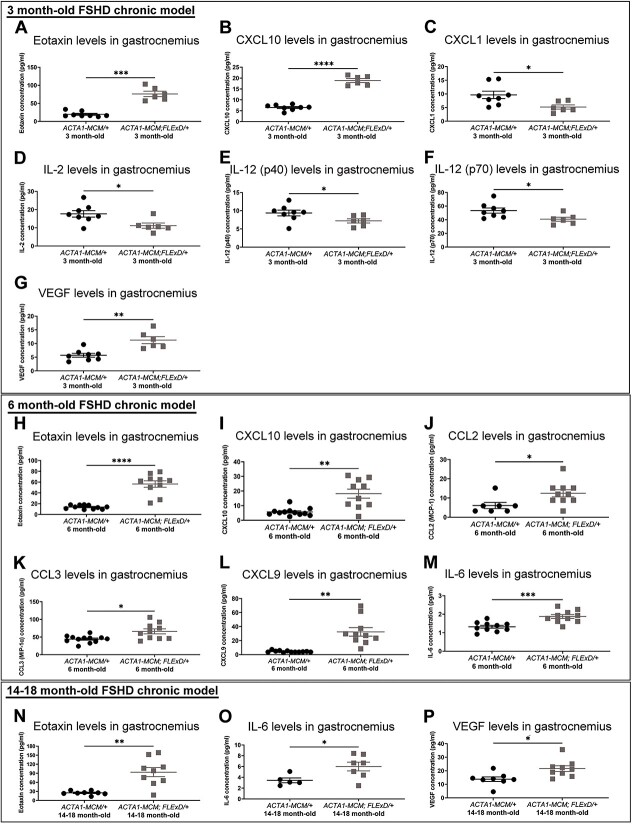
Cytokine/chemokine profile in the skeletal muscle from chronic FSHD-like mice. Luminex protein quantification of cytokines/chemokines (A) eotaxin, (B) CXCL10, (C) CXCL1, (D) IL-2, (E) IL-12 (p40), (F) IL-12 (p70), and (G) VEGF in the gastrocnemius muscle of 3 month-old chronic FSHD-like mice. Luminex protein quantification of cytokines/chemokines (H) eotaxin, (I) CXCL10, (J) CCL2, (K) CCL3, (L) CXCL9, and (M) IL-6 in the gastrocnemius muscle of 6 month-old chronic FSHD-like mice. Luminex protein quantification of cytokines/chemokines (N) eotaxin, (O) IL-6, and (P) VEGF in the gastrocnemius muscle of 14–16 month-old chronic FSHD-like mice. Statistical analysis was performed using two-tailed Welch’s t-test. The numbers (n) are indicated as individual points in each graph. Protein levels below the detection threshold were removed from the analysis. Data are presented as mean ± s.e.m.; ^*^P < 0.05, ^*^^*^P < 0.01, ^*^^*^^*^^*^P < 0.0001. Alt text: Greyscale figure with scatter plots showing cytokine/chemokine profiles from FSHD chronic mouse models.

To further characterize the immune response to skeletal muscle expression of DUX4, we performed a cytokine/chemokine profile of the gastrocnemius muscle from 3, 6 and 14–18 month-old FSHD-like chronic mice (Fig. 1 and Tables S1–S3). Strikingly, we found that levels of eotaxin, also known as CCL11, were significantly increased by 4-fold in gastrocnemius muscles from all ages of *ACTA1-MCM/FLExD/+* compared to *ACTA1-MCM/+* aged-matched controls (Fig. 1A, H and N). Other notable differences include the increase of CXCL10 levels in muscles from 3 and 6 month-old *ACTA1-MCM/FLExD/+* mice (Fig. 1B and I), and the increase of IL-6 levels in muscles from 6 month-old (Fig. 1M) and 14–18 month-old *ACTA1-MCM/FLExD/+* mice (Fig. 1O). In addition, levels of CXCL1 (Fig. 1C), IL-2 (Fig. 1D) and IL-12 (p40), IL-12 (p70) (Fig. 1E and F) were significantly decreased in the muscle of 3 month-old *ACTA1-MCM/FLExD/+* mice compared to age-matched controls. In contrast, the levels of VEGF were increased in the muscle of 3 month-old *ACTA1-MCM/FLExD/+* mice compared to age-matched controls (Fig. 1G). At 6 months of age, the muscle from *ACTA1-MCM/FLExD/+* mice also displayed increased expression of CCL2, CCL3, and CXCL9 (Fig. 1J–L). The levels of most cytokine/chemokines were below the detection threshold or unchanged in the muscles from 14–18 month-old *ACTA1-MCM/FLExD/+* mice when compared to controls (Table S3). Apart from eotaxin and IL-6, we found VEGF to be the only other protein with significantly increased expression in 14–18 month-old *ACTA1-MCM/FLExD/+* mice compared to aged-matched controls (Fig. 1P). These results showed that while DUX4-induced changes in the levels of many cytokines/chemokines in skeletal muscle are dynamic throughout different stages of disease progression, expression of eotaxin is consistently elevated in DUX4 expressing skeletal muscles at all stages of disease progression.

### Chemokine enrichment in the serum of chronic FSHD-like mice

**Figure 2:**
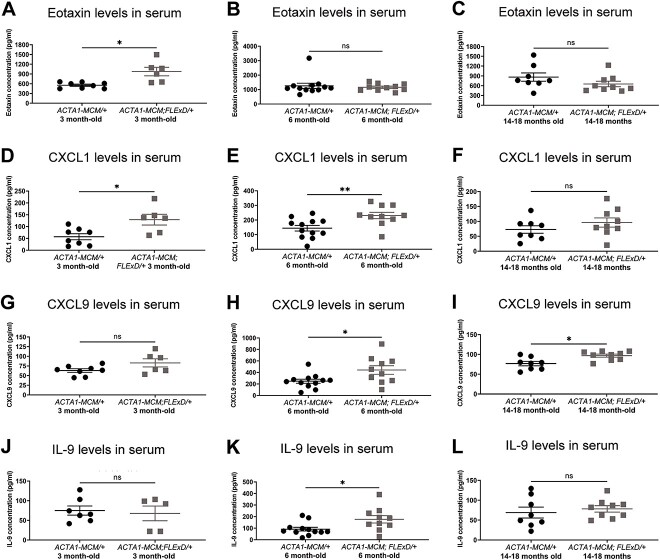
Cytokine/chemokine profile in the serum from chronic FSHD-like mice. Luminex protein quantification of cytokines/chemokines (A–C) eotaxin, (D–F) CXCL1, (G–I) CXCL9, and (J–L) IL-9 in the serum from 3, 6 and 14–18 month-old chronic FSHD-like mice. Statistical analysis was performed using two-tailed Welch’s t-test. The numbers (n) are indicated as individual points in each graph. Protein levels below the detection threshold were removed from the analysis. Data are presented as mean ± s.e.m.; ^*^P < 0.05, ^*^^*^P < 0.01, ^*^^*^^*^^*^P < 0.0001. Alt text: Greyscale figure with scatter plots depicting cytokine/chemokine serum levels in mice.

To evaluate the cytokine/chemokine expression profile, we analyzed the serum of 3, 6 and 14–18 month-old FSHD-like chronic mice (Fig. 2). We found that the levels of most cytokine/chemokines in these mice were below the detection threshold or unchanged in *ACTA1-MCM/FLExD/+* animals compared to the respective *ACTA1-MCM/+* age-matched controls (Tables S4–S6). We verified that eotaxin levels were elevated in 3 month-old *ACTA1-MCM/FLExD/+* animals, but unchanged in 6 and 14–18 month-old animals compared to age-matched controls (Fig. 2A–C, Tables S4–S6). We also found that the levels of CXCL1 were increased in 3 and 6 month-old *ACTA1-MCM/FLExD/+* animals, but similar in 14–18 month-old *ACTA1-MCM/FLExD/+* when compared to age-matched control animals (Fig. 2D–F, Tables S4–S6). In contrast, the levels of CXCL9 are unchanged in the serum of 3 month-old *ACTA1-MCM/FLExD/+* animals (Fig. 2G) and elevated in the serum of 6 and 14–18 month-old *ACTA1-MCM/FLExD/+* animals compared to *ACTA1-MCM/+* controls (Fig. 2H and I). The levels of muscle CXCL9 are only elevated in 6 month-old *ACTA1-MCM/FLExD/+* animals (Fig. 1L). We found that IL-9 expression was elevated in the serum of 6-month-old *ACTA1-MCM/FLExD/+* animals (Fig. 2K) and unchanged in the serum of 3 and 14–18 month-old animals (Fig. 2J and L). Together, these results indicate that the serum from FSHD-like chronic mice was enriched with chemokines and cytokines, particularly eotaxin at early stages, CXCL1 at early and mid-stages, and CXCL9 at mid and late stages of disease progression.

**Figure 3:**
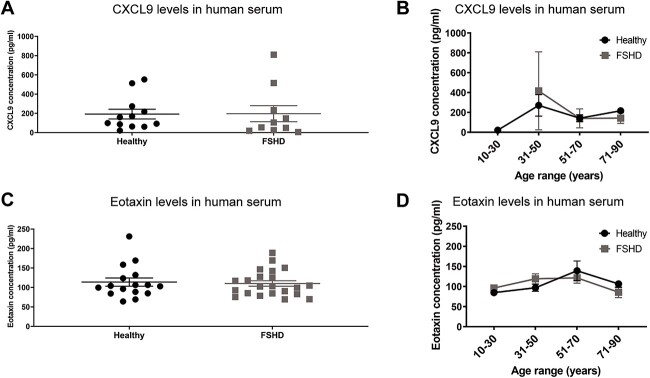
Eotaxin and CXCL9 expression in human serum. Expression of (A) CXCL9 and (C) eotaxin in human serum from all FSHD and healthy subjects. Expression of (B) CXCL9 and (D) eotaxin in human serum according to patient/subject age. Statistical analysis was performed using two-tailed Welch’s t-test with n = 22 FSHD subjects and n = 16 healthy subjects for eotaxin analysis and two-tailed Mann-Whitney test with n = 10 FSHD subjects and n = 12 healthy- subjects for CXCL9 analysis. Protein levels below the detection threshold were removed from the analysis. Data are presented as mean ± s.e.m.; ^*^P < 0.05, ^*^^*^P < 0.01, ^*^^*^^*^^*^P < 0.0001. Alt text: Greyscale figure with scatter plots of healthy vs FSHD human data on CXCL9 and eotaxin serum levels either combined (A and C) or separated by age ranges (B and D).

To further evaluate if CXCL9 and eotaxin are candidate FSHD circulating biomarkers, we quantified the levels of these chemoattractants in the sera of FSHD patients and healthy subjects through ELISA. However, we found no significant changes in the expression of CXCL9 or eotaxin in the serum from FSHD patients compared to healthy subjects (Fig. 3A and C). We further analyzed the study cohort stratified by age and found no differences in the expression levels of CXCL9 or eotaxin (Fig. 3B and D). These results suggest that CXCL9 and eotaxin might each reflect different stages of disease progression in the mouse model, but are not prime candidates for circulating biomarkers for the human disease.

### The eosinophil population is enriched in the muscle of chronic FSHD-like mice

**Figure 4:**
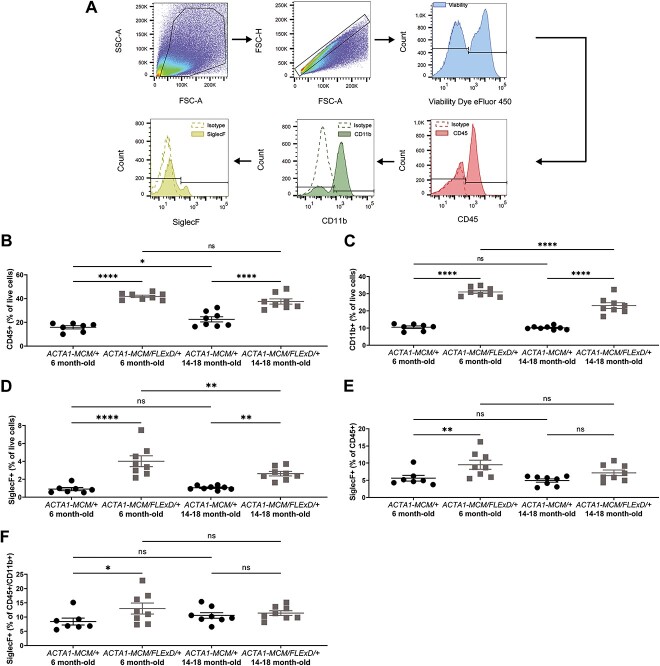
Flow cytometry analysis of skeletal muscle in chronic FSHD-like mice. Gating strategy with selection of live high FSC-A and SSC-A singlets and staining for (A) CD45, CD11b and SiglecF. Leukocytes were defined as CD45^+^, myeloid cells as CD45^+^CD11b^+^ and eosinophils as CD45^+^CD11b^+^SiglecF^+^. (B) Quantification of CD45^+^ cell percentage from live cells. (C) Quantification of CD11b^+^ cell percentage from live cells. Quantification of eosinophil SiglecF^+^ percentage from (D) live cells, (E) CD45^+^ cells, and (F) CD45^+^CD11b^+^ cells. Statistical analysis was performed using one-way ANOVA with n = 7 for 6 month-old *ACTA1-MCM/+*, n = 8 for 6 month-old *ACTA1-MCM/FLExD/+*, n = 8 for 14–18 month-old *ACTA1-MCM/+* and n = 8 for 14–18 month-old *ACTA1-MCM/FLExD/+*. Data are presented as mean ± s.e.m.; ^*^P < 0.05, ^*^^*^P < 0.01, ^*^^*^^*^^*^P < 0.0001. Alt text: Panel A shows FACS histograms depicting the gating strategy for cell counting. Panels B-F are greyscale scatter plot graphs.

To identify cells involved in the immune response in the chronic FSHD-like mouse model, we designed a flow cytometry panel and a gating strategy based in part on our cytokine results (Fig. 4A). We focused our analysis on the myeloid/eosinophil population considering that our cytokine profile revealed an increase of chemoattractants/cytokines such as CXCL9, CXCL10, CCL2, and CCL3, which are known to attract myeloid cells [36–40]. More importantly, eotaxin is a specific eosinophil chemoattractant [41–43] and we found that eotaxin expression was increased at all stages of disease in the muscle of chronic FSHD-like mice (Fig. 1A, H and N). We used established markers for the detection of leukocytes (CD45^+^), myeloid cells (CD45^+^CD11b^+^), and eosinophils (CD45^+^ CD11b^+^ and SiglecF^+^) in skeletal muscle [44]. The markers and protocol were validated in skeletal muscle isolated from *mdx* mice, a model of Duchenne muscular dystrophy with well-characterized muscle immune infiltration [45–47] (Fig. S2).

Analysis of the gastrocnemius muscle from chronic FSHD-like mice revealed that the leukocyte CD45^+^ population was enriched by 3- and 2-fold in 6 and 14–18 month-old *ACTA1-MCM/FLExD/+* muscles, respectively, when compared to *ACTA1-MCM/+* age-matched controls (Fig. 4B). Interestingly, the enrichment in immune infiltrates appears to plateau at ~6 months, as demonstrated by the similar amount of immune muscle infiltrates in 6 and 14–18 month-old *ACTA1-MCM/FLExD/+* mice (Fig. 4B). We found that part of the leukocyte CD45^+^ influx into the muscle was associated with a significant increase in the myeloid population (CD45^+^CD11b^+^) in 6 and 14–18 month-old *ACTA1-MCM/FLExD/+* muscles (Fig. 4C). Further quantification of eosinophils (CD45^+^CD11b^+^SiglecF^+^) revealed an increase of 4- and 2-fold in 6 and 14–18 month-old *ACTA1-MCM/FLExD/+* muscles, respectively, compared to age-matched controls (Fig. 4D). To determine if the eosinophil influx into the DUX4 expressing muscles was a consequence of the overall increase in leukocyte infiltration and/or an increase of the eosinophil sub-population, we quantified eosinophils within the leukocyte CD45^+^ and the myeloid CD45^+^CD11b^+^ populations (Fig. 4E and F). We found that the numbers of eosinophils within the CD45^+^ leukocyte and the CD45^+^CD11b^+^ myeloid populations were elevated by 2-fold in chronic 6 month-old *ACTA1-MCM/FLExD/+* muscles compared to age-matched controls. In contrast, the proportion of eosinophils within the leukocyte and myeloid populations was similar in 14–18 month-old *ACTA1-MCM/FLExD/+* and *ACTA1-MCM/+* age-matched control muscles (Fig. 4E and F). Additional hematoxylin and eosin (H&E) staining of 6 month-old (Fig. S3A, A’, B and B’) and 14–18 month-old (Fig. S3C, C’, D and D’) gastrocnemius muscle revealed the presence of granulated leukocytes, potentially eosinophils.

Together, these results support that chronic expression of DUX4 in skeletal muscle is associated with increased immune infiltration, even though this enrichment did not increase further with age. Additionally, these results suggested that the immune cells infiltrating DUX4 expressing muscles are composed of myeloid cells, particularly eosinophils. Importantly, the immune infiltration in mid-stages of the disease (i.e. 6 months of age) is characterized at least by a specific eosinophil driven infiltration.

### Circulating immune cells are not affected in chronic FSHD-like mice

**Figure 5:**
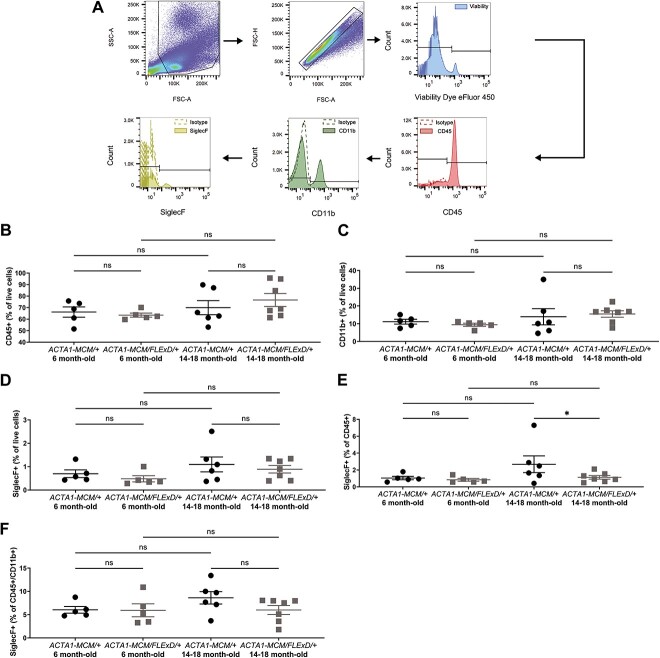
Flow cytometry analysis of blood from chronic FSHD-like mice. Gating strategy with selection of live high FSC-A singlets and staining for (A) CD45, CD11b and SiglecF. Leukocytes were defined as CD45^+^, myeloid cells as CD45^+^CD11b^+^ and eosinophils as CD45^+^CD11b^+^SiglecF^+^. (B) Quantification of CD45^+^ cell percentage from live cells. (C) Quantification of CD11b^+^ cell percentage from live cells. Quantification of eosinophil SiglecF^+^ percentage from (D) live cells, (E) CD45^+^ cells, and (F) CD45^+^ CD11b^+^ cells. Statistical analysis was performed using one-way ANOVA with n = 5 for 6 month-old *ACTA1-MCM/+*, n = 5 for 6 month-old *ACTA1-MCM/FLExD/+*, n = 6 for 14–18 month-old *ACTA1-MCM/+* and n = 7 for 14–18 month-old *ACTA1-MCM/FLExD/+*. Data are presented as mean ± s.e.m.; ^*^P < 0.05, ^*^^*^P < 0.01, ^*^^*^^*^^*^P < 0.0001. Alt text: Panel A shows FACS histograms depicting the gating strategy for cell counting. Panels B-F are greyscale scatter plot graphs.

We employed the same experimental design and detection panel described above for skeletal muscle to investigate any potential changes in the composition of circulating immune cells by flow cytometry (Fig. 5A). We found that the proportion of CD45+ leukocytes (Fig. 5B), CD45 + CD11b + myeloid cells (Fig. 5C), and CD45^+^CD11b^+^SiglecF^+^ eosinophils (Fig. 5D–F) was similar in 6 and 14–18 month-old *ACTA1-MCM/FLExD/+* mice compared to age-matched *ACTA1-MCM/+* controls. These data indicated that no significant changes occurred in the pool of circulating immune cells and therefore support that the immune cell influx into DUX4-expressing muscles likely represents a local response to DUX4 expression.

### FSHD muscle is enriched with the eosinophil peroxidase

**Figure 6:**
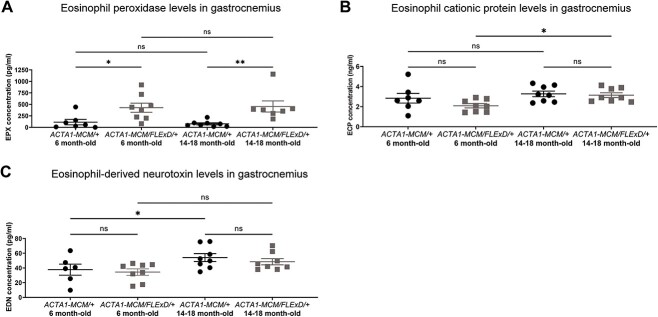
Eosinophil activation in the skeletal muscle of chronic FSHD-like mice. Quantification of (A) eosinophil peroxidase (EPX), (B) eosinophil cationic protein (ECP), and (C) eosinophil-derived neurotoxin (EDN). Statistical analysis was performed with one-way ANOVA. EPX was quantified using n = 6 for 6 month-old *ACTA1-MCM/+*, n = 8 for 6 month-old *ACTA1-MCM/FLExD/+*, n = 8 for 14–18 month-old *ACTA1-MCM/+* and n = 7 for 14–18 month-old *ACTA1-MCM/FLExD/+*. ECP was quantified using n = 6 for 6 month-old *ACTA1-MCM/+*, n = 8 for 6 month-old *ACTA1-MCM/FLExD/+*, n = 8 for 14–18 month-old *ACTA1-MCM/+* and n = 8 for 14–18 month-old *ACTA1-MCM/FLExD/+*. EDN was quantified using n = 7 for 6 month-old *ACTA1-MCM/+*, n = 8 for 6 month-old *ACTA1-MCM/FLExD/+*, n = 8 for 14–18 month-old *ACTA1-MCM/+* and n = 8 for 14–18 month-old *ACTA1-MCM/FLExD/+*. Data are presented as mean ± s.e.m.; ^*^P < 0.05, ^*^^*^P < 0.01, ^*^^*^^*^^*^P < 0.0001. Alt text: Greyscale scatter plots of the data from murine gastrocnemius muscle.

**Figure 7:**
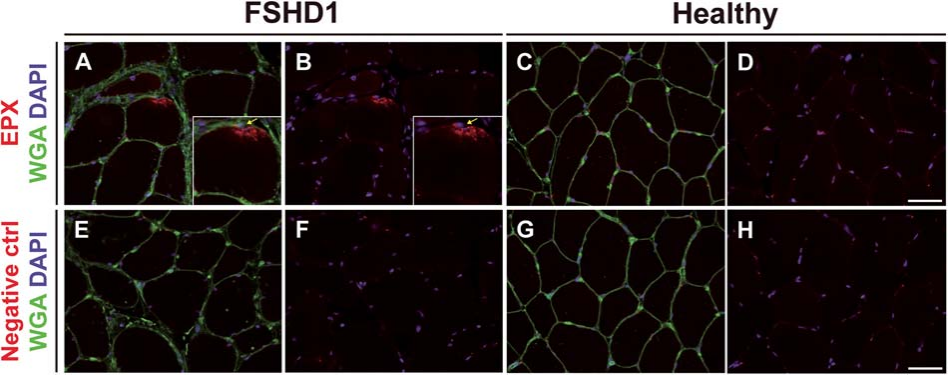
Eosinophil peroxidase expression in human skeletal muscle biopsies. Immunofluorescence for eosinophil peroxidase (EPX), wheat germ agglutinin (WGA), and 4′,6-diamidino-2-phenylindole (DAPI) in human muscle biopsies from (A, B, E and F) FSHD and healthy (C, D, G and H) patients. Negative control (negative ctrl) staining without primary antibody (E–H) is presented to clarify the positive staining with anti-EPX antibody (A–D). The yellow arrow indicates a nucleus close to the EPX positive staining. n = 3 FSHD1 muscle biopsies and n = 4 healthy muscle biopsies. Scale bar: 50 μm. Alt text: Microscopy images of histological sections of FSHD1 and healthy muscle stained for EPX, WGA, and DAPI.

Muscle eosinophilia should lead to an increase of eosinophil cytotoxic proteins; thus, we quantified the expression levels of these from the gastrocnemius muscle of 6 and 14–18 month-old chronic FSHD-like mice (Fig. 6). Although there were no significant differences in the muscle expression of eosinophil cationic protein (ECP) and eosinophil-derived neurotoxin (EDN), the expression of eosinophil peroxidase (EPX) was significantly elevated by 4- and 6-fold in 6 and 14–18 month-old *ACTA1-MCM/FLExD/+* muscles, respectively, compared to age-matched controls (Fig. 6). To further evaluate EPX expression in human muscle biopsies, we stained gastrocnemius sections from FSHD1 and healthy subjects (Fig. 7). We identified muscle fibers with positive EPX staining in 2 out of 3 FSHD biopsies (Fig. 7A, B, E and F and Fig. S4A and C) in a pattern similar to the one previously described [48], whereas no EPX positive fibers were detected in the 4 healthy biopsies (Fig. 7C, D, G and H and Fig. S4B, D and E). These results suggest that EPX might be an important determinant of eosinophil activity in skeletal muscle of both chronic FSHD-like mice and FSHD subjects.

## Discussion

Chronic inflammation is known to have a negative impact on the pathological progression of the muscular dystrophies. The current knowledge supports the notion that FSHD pathology is not driven by autoantibody reactivity against muscle antigens [25]. However, FSHD pathology is clearly associated with the infiltration of both B and T lymphocytes and macrophages [18] as well as the activation of the complement signaling pathway [23, 24]. Here, we characterize the immune infiltration in the chronic FSHD-like mouse model of DUX4 expression. We demonstrate that muscles with chronic expression of DUX4 are enriched with eotaxin, a major eosinophil chemoattractant, and EPX. DUX4 expressing muscles also exhibit an increase in the muscle influx of eosinophils. We propose muscle eosinophilia as a new immune-related pathology marker of FSHD.

### Muscle eosinophilia in FSHD and muscular dystrophies

Eosinophils are granular terminally differentiated leukocytes derived from the bone marrow [49]. Eosinophil granules are composed of cytotoxic proteins, such as the major basic protein-1 (MBP-1), EPX, ECP, and EDN, which eosinophils release upon immune activation [49–51]. They are critical effectors of the type 2 inflammatory response with a long-standing association with multiple diseases including allergic rhinitis, asthma eosinophilic, chronic rhinosinusitis, and esophagitis [49, 52]. Muscle eosinophilia has been reported in several neuromuscular diseases including Duchenne and Becker Muscular Dystrophy [53–56], *LAMA2*-CMD muscular dystrophy [57], Limb-girdle muscular dystrophy [58, 59], and idiopathic eosinophilic myositis [60, 61]. However, the only study to date that quantified eosinophils in FSHD reported an increase in the hemogram eosinophil count in 1 out of 3 FSHD patients analyzed [62]. In that study, eosinophil quantification by giemsa and H&E staining demonstrated that eosinophil numbers in FSHD patient muscles do not significantly differ from those in control subject muscles [62].

Our study demonstrates that chronic expression of DUX4 in skeletal muscle causes muscle immune cell infiltration composed of a myeloid sub-population of eosinophils. In mid-stages of disease progression, muscle eosinophilia reflects a shift toward a specific infiltration of eosinophils and production of EPX, whereas the eosinophil muscle influx in later stages of the disease is solely associated with an overall increase in the leukocyte recruitment to the muscle. Thus, it seems likely that eosinophils play a mid-stage role during disease progression in FSHD. In accordance, we detected EPX positive fibers in 2 out of 3 FSHD patient muscle biopsies. Several studies support a role for eosinophils in the regulation of fibrosis and adipogenesis [54, 63], particularly in the *mdx* mouse model of DMD where chronic muscle eosinophilia contributes to fibrosis [54, 55, 64, 65]. Previous studies on the *mdx* mouse model revealed that eosinophils induce cell lysis *in vivo* in a MBP-1-independent mechanism, whereas muscle fibrosis is dependent on MBP-1 [64]. While we were unable to successfully quantify the muscle levels of MBP-1 in the chronic FSHD-like mouse model (data not shown), we suggest that EPX may have an important role in FSHD pathology as an effector of eosinophil-mediated fibrosis. We speculate that eosinophil recruitment to the muscle might represent a common myeloid response to chronic exposure to muscle damage patterns, a common marker and general mechanism in muscular dystrophies with different etiologies.

### Blood eosinophils and eotaxin in FSHD and muscular dystrophies

Eotaxin is a major eosinophil chemoattractant that induces eosinophil release from the bone marrow and recruitment to tissues [41, 43, 66]. We show that DUX4 expression induces a localized muscle immune response characterized by increased eotaxin expression and eosinophil recruitment. Chronic expression of DUX4 in mice did not induce changes in the blood eosinophil composition in the mid and late stages of FSHD progression, suggesting that changes in eosinophil release from the bone marrow are unlikely to happen at these stages. Interestingly, muscle eosinophilia without blood eosinophilia seems to be rather common in muscular dystrophies. For example, muscle eosinophilia in the *dyW* mouse model of *LAMA2*-CMD [57] correlates with increased eotaxin expression in the skeletal muscle in *dyW* mice [57, 67, 68], although eotaxin levels in the serum of *dyW* mice are normal [67]. Proteomic analysis of human muscle biopsies also revealed a significant increase in eotaxin expression in *LAMA2*-CMD patients [67]. Similarly, muscle eosinophilia is evident in the *mdx* mouse model of Duchenne Muscular Dystrophy [53, 65], where serum levels of eotaxin are normal [65]. Taken together, our study supports a model whereby DUX4 expression directly or indirectly induces the local expression of eotaxin and other chemokines in immune cells and/or muscle fibers as a general damage signal that further leads to leukocyte recruitment.

In accordance with this model, our analysis of human serum samples revealed no changes in eotaxin levels in FSHD patients and excluded eotaxin as a potential circulating biomarker for FSHD. However, considering the enrichment of eotaxin in the muscle of chronic FSHD-like mice at different stages of disease, we propose that eotaxin might still be validated as a muscle biomarker for the human disease. The successful implementation of muscle fluid collection from FSHD patients [69] provides a promising option for the future analysis of eotaxin in FSHD human muscle in the clinic.

Overall, we characterized a new aspect of immune infiltration in the DUX4-expressing chronic FSHD-like mouse model. We identified muscle eosinophilia as a new immune-related marker of FSHD pathology and demonstrate that muscle eosinophilia correlates with an increase in the expression of eotaxin and EPX in muscle. Thus, our findings in the FSHD-like mouse model are consistent with the limited clinical data, supporting eosinophilia as a potential aspect of FSHD pathology. Future human studies will be important to determine the role of muscle eosinophilia during disease progression and its validation as a biomarker.

## Materials and methods

### Animals

*ACTA1-MCM* [28] and *ACTA1-MCM; FLExD* mice were bred in our laboratory and genotyped as previously described [27]. *ACTA1-MCM* and *ACTA1-MCM; FLExD* mice were aged until 3, 6 and 14–18 months old [26]. Mice were sacrificed under deep anesthesia (3% isoflurane).

### Luminex xMAP® immunoassay

Muscle homogenates were prepared from freshly dissected gastrocnemius muscle as previously described [70] with the sample-grinding kit (GE Healthcare Bio-Sciences, 80-6483-37), using half the volume of buffer recommended by the manufacturer [70]. Muscle homogenate samples were normalized to a concentration of 2000 μg/ml. Blood was centrifuged at 1000 × g at 15°C for 10 min or 15 min for mouse and human samples, respectively, and serum was collected. Luminex xMAP immunoassay was performed in the University of California, Los Angeles Immune Assessment Core. The mouse 32-plex magnetic cytokine/chemokine kit was purchased from EMD Millipore and used per the manufacturer’s instructions. Briefly, 25 μl undiluted (or for mouse, 1:2 diluted) samples were mixed with 25 μl magnetic beads, and allowed to incubate overnight at 4°C while shaking. After washing the plates twice with wash buffer in a Biotek ELx405 washer, 25 μl of biotinylated detection antibody was added and incubated for 1 h at room temperature. Streptavidin-phycoerythrin conjugate (25 μl) was then added to the reaction mixture and incubated for another 30 min at room temperature. Following two washes, beads were resuspended in sheath fluid, frozen. Fluorescence was quantified using a Luminex 200TM instrument.

### Muscle leukocyte isolation

Leukocyte isolation from the hindlimb (pool of gastrocnemius, *extensor digitorum longus*, quadriceps and *tibialis anterior* muscles) and triceps muscles was performed as previously described [44]. Briefly, the muscles were dissected and kept in cold DMEM (Dulbecco’s Modified Eagle’s Medium) (Corning, 10-013-CV) with 10% FBS (fetal bovine serum) (Hyclone/Cytiva SH30396.03). The muscles were then minced, transferred to 10 ml of digestion media (DMEM with 0.2 mg/ml collagenase P (Roche, Sigma, 11213873001) and 0.02 mg/ml DNAse I (Roche, Sigma, 10104159001)) and incubated for one hour at 37°C. The resulting cell suspension was strained in 100, 70 and 40 μm strainers and separated in the Histopaque-1077 gradient (Sigma, H8889) to isolate the immune cells. The cell suspension was resuspended in 1X PBS/2% FBS.

### Blood leukocyte isolation

Blood leukocyte isolation was performed as previously described [71]. Collection of 500 μl of blood was performed in EDTA-coated tubes (Microvette® 200 K3 EDTA, Cat. 20.1288.100). Blood samples were incubated with 2 ml of 1× red blood cell lysis (diluted from 10×, Cat. sc-296258) for 15 min at room temperature and then centrifuged for 5 min at 500 × g. The pellet was resuspended with 2 ml of 1× red blood cell lysis, incubated for additional 15 min and centrifuged for 5 min at 500 × g. The cell pellet was then resuspended in 1X PBS/2% FBS.

### Staining for flow cytometry

The cells were incubated with TruStain FcX™ (anti-mouse CD16/32) Antibody (Biolegend, 101319) for 15 min on ice and then stained for 30 min on ice with the following antibodies: CD45 APC-Cy7 (1:20; clone 30-F11, BD Biosciences, Cat. 561037), Cd11b FITC (1:20, clone M1/70, BD Biosciences, Cat. 561688) and SiglecF PE (1:20; clone E50-2440, BD Biosciences, Cat. 562068). Viability staining was performed with the eBioscience™ Fixable Viability Dye eFluor™ 450 (Cat. 65-0863-14) for 30 min on ice according to the manufacturer’s instructions.

### Protein quantification by ELISA

ELISA analysis was performed according to the manufacturer’s instructions: mouse MHC I (Abbexa, Cat. abx254262), mouse eosinophil peroxidase (LsBio, Cat. LS-F7707-1), mouse eosinophil-derived neurotoxin (Biomatik, Cat. EKC42359-96T), mouse major basic protein (antibodies-online.com, Cat. ABIN6720571), mouse eosinophil cationic protein (Biomatik, Cat. EKC40815), human eotaxin (ThermoFisher, Cat. KAC2231) and CXCL9 (Abcam, Cat. ab119588).

### Immunostaining

Sections from human gastrocnemius muscle were fixed and permeabilized with acetone at −20°C for 5 min. Sections were blocked with 5% normal donkey serum and 5% bovine serum albumin for 1 h at room temperature and then incubated overnight at 4°C with anti-EPX antibody (1:100; Sigma Cat. HPA050507). Sections were incubated for 45 min with anti-rabbit secondary antibody (1:1000; Jackson ImmunoResearch Cat. 711-586-152) and then incubated with wheat germ agglutinin (1:500; ThermoFisher Cat. W32466) for 5 min.

### Statistical analysis

Normality tests were performed for each data set to determine the appropriate parametric or non-parametric statistical test. The statistical analysis is indicated in each figure legend. The statistical analysis was performed with Graphpad Prism 8. Statistical significance was considered as following: ^*^P < 0.05, ^*^^*^P < 0.01, ^*^^*^^*^P < 0.001, ^*^^*^^*^^*^P < 0.0001.

### Study approval

All procedures involving animals were approved by the University of Nevada, Reno IACUC (protocols #0701 and #20-09-1078). All procedures involving human patients were approved by the University of Nevada, Reno Institutional Review Board (#1316095). This clinical investigation was conducted according to the principles expressed in the Declaration of Helsinki. Written informed consent was received prior to participation. All patient sera were de-identified and processed in a randomized and blinded manner.

## Conflict of interest statement

The authors declare no conflicts of interest.

## Funding

We thank Dr Charis L. Himeda for helpful discussions and editing the manuscript. We thank Dr Rabi Tawil and Don Henderson, University of Rochester Medical Center, for providing the FSHD and healthy muscle sections for immunohistochemistry. This work was supported by grant R01AR062587 from the National Institute of Arthritis and Musculoskeletal and Skin Diseases, National Institutes of Health, to P.L.J. and a grant from the FSHD Canada Foundation to AMN. P.L.J. is supported by the Mick Hitchcock, PhD Endowed Chair in Medical Biochemistry at the University of Nevada, Reno School of Medicine.

## Data availability

All data will be available upon request.

## Supplementary Material

Supplementary_figure_1_ddae019

Supplementary_figure_2_ddae019

Supplementary_figure_3_ddae019

Supplementary_figure_4_ddae019

Supplementary_Table_1_ddae019

Supplementary_Table_2_ddae019

Supplementary_Table_3_ddae019

Supplementary_Table_4_ddae019

Supplementary_Table_5_ddae019

Supplementary_Table_6_ddae019
