# Supplementary material for: Muscle eosinophilia is a hallmark of chronic disease in facioscapulohumeral muscular dystrophy
Source: Hum Mol Genet. 2024 Feb 10;33(10):872–83. doi: 10.1093/hmg/ddae019 (PMC11070135; doi:10.1093/hmg/ddae019)
Supplement: Supplementary_figure_1_ddae019 [file supplementary_figure_1_ddae019.pdf]

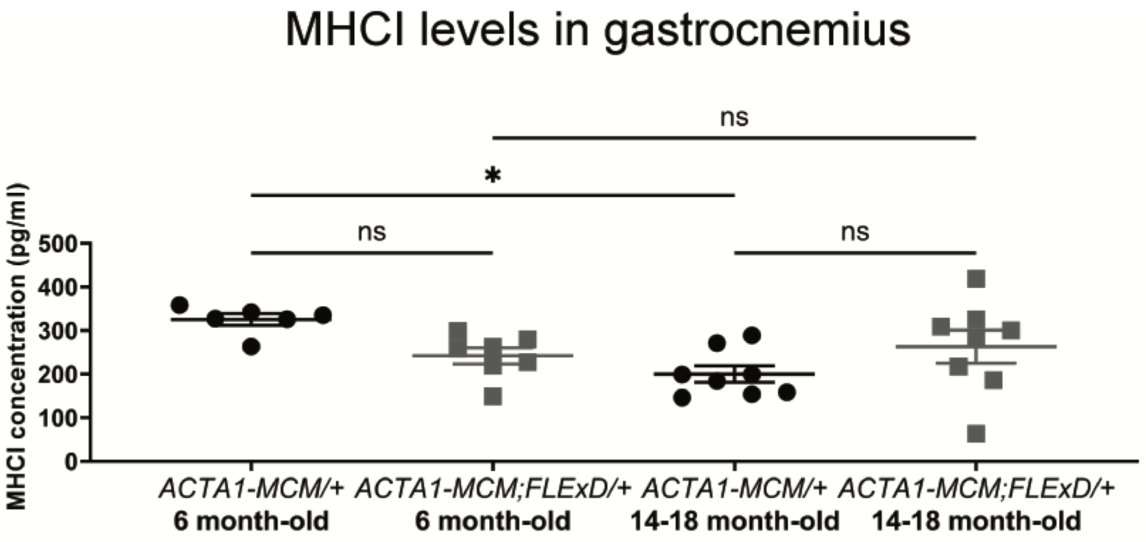

Figure S1- Major histocompatibility complex I in the skeletal muscle of chronic FSHD-like mice. Protein quantification of MHC I in the gastrocnemius muscle from 6 and 14-16 months-old FSHD chronic mice. Statistical analysis was performed with one-way ANOVA with n=6 for 6 month-old *ACTA1-MCM/+*, n=7 for 6 months-old *ACTA1-MCM/FLExD/+*, n=8 for 14-18 month-old *ACTA1-MCM/+* and n=8 for 14-18 months-old *ACTA1-MCM/FLExD/+*. Data are presented as mean ±s.e.m.; *P<0.05, **P<0.01, ****P<0.0001.

Alt text: Greyscale scatter plots showing protein quantification of MHC complex I in gastrocnemius muscle of 6 14-18 month-old mice.
