# Supplementary material for: Muscle eosinophilia is a hallmark of chronic disease in facioscapulohumeral muscular dystrophy
Source: Hum Mol Genet. 2024 Feb 10;33(10):872–83. doi: 10.1093/hmg/ddae019 (PMC11070135; doi:10.1093/hmg/ddae019)
Supplement: Supplementary_figure_2_ddae019 [file supplementary_figure_2_ddae019.pdf]

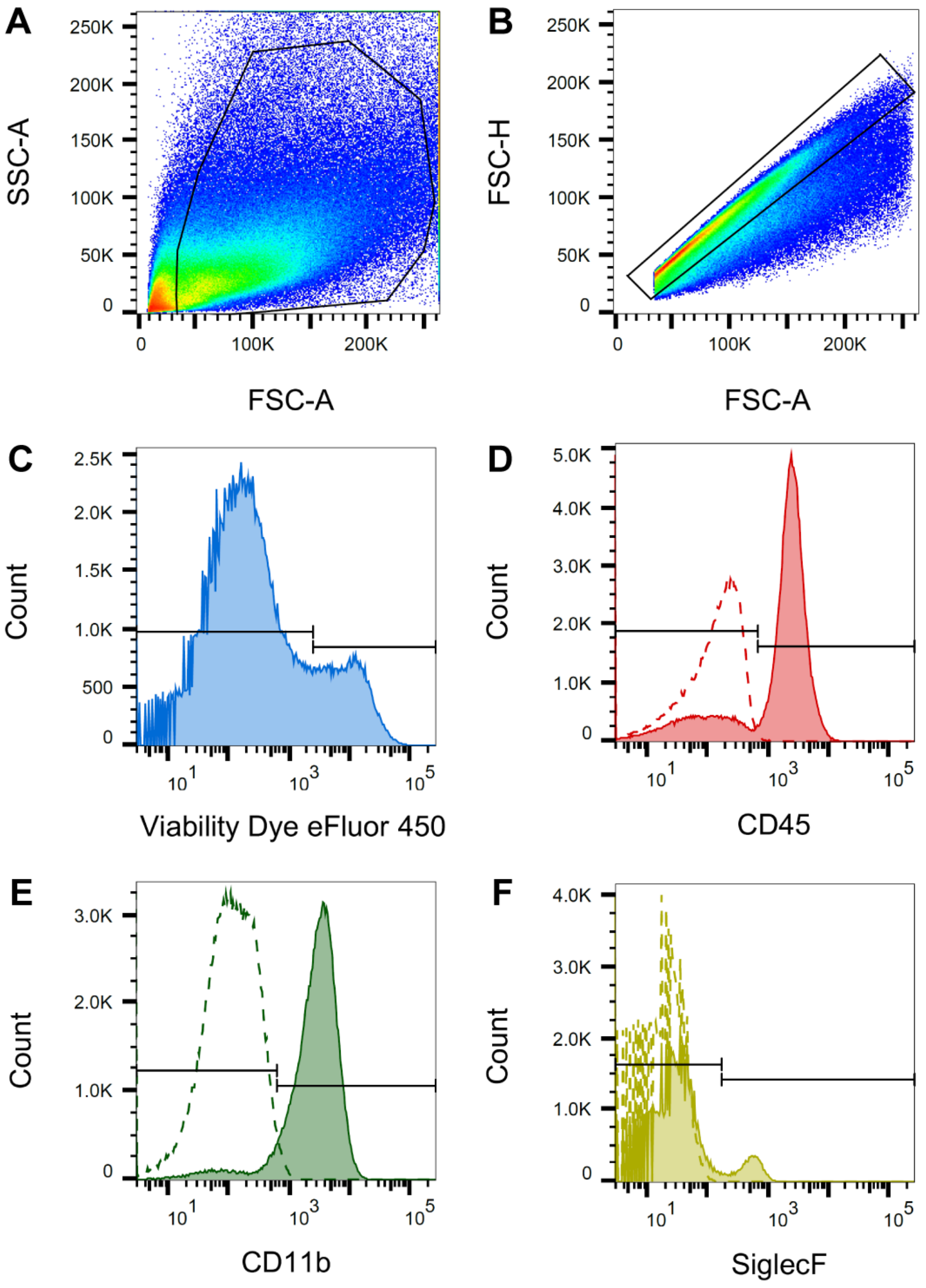

Figure S2- Flow cytometry antibody validation in the skeletal muscle of *mdx* mouse model. Immune cells were analyzed in the cell gate with (A) higher FSC-A and SSC-A and (B) in the singlets sub-population. (C) Viability Dye eFluor 450 staining was used to select the live dye negative cells. Analysis on live cells for (D) CD45, (E) CD11b, (F) and SiglecF.

Alt Text: Full-color histograms of FACs validation data from *mdx* mice.
