# Supplementary material for: Muscle eosinophilia is a hallmark of chronic disease in facioscapulohumeral muscular dystrophy
Source: Hum Mol Genet. 2024 Feb 10;33(10):872–83. doi: 10.1093/hmg/ddae019 (PMC11070135; doi:10.1093/hmg/ddae019)
Supplement: Supplementary_figure_3_ddae019 [file supplementary_figure_3_ddae019.pdf]

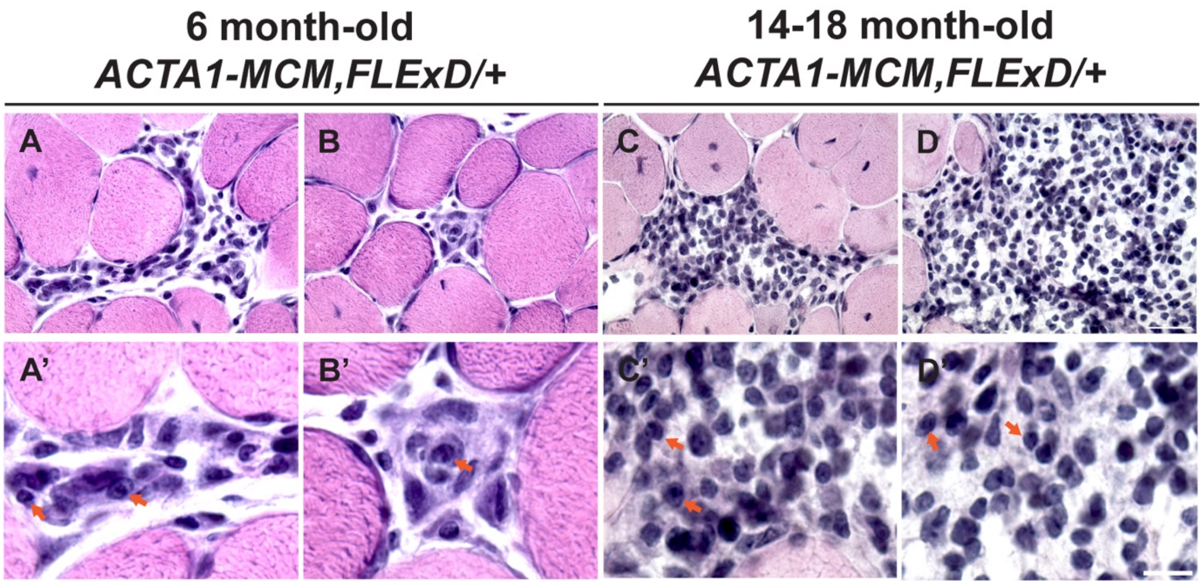

Figure S3- Hematoxylin and eosin staining in the gastrocnemius muscle of chronic FSHD-like mice. Immune infiltrates in the gastrocnemius muscle of 6 month-old *ACTA1-MCM; FLExD/*+ mice (A, A', B, B'). Immune infiltrates in the gastrocnemius muscle of 14-18 month-old *ACTA1-MCM; FLExD/*+ mice (C, C', D, D'). Potential eosinophils are indicated with orange arrows. Scale: 25 µm for D. Scale: 10 µm for D'.

Alt text: Color figures showing H&E staining of muscle sections.
