# Supplementary material for: Muscle eosinophilia is a hallmark of chronic disease in facioscapulohumeral muscular dystrophy
Source: Hum Mol Genet. 2024 Feb 10;33(10):872–83. doi: 10.1093/hmg/ddae019 (PMC11070135; doi:10.1093/hmg/ddae019)
Supplement: Supplementary_figure_4_ddae019 [file supplementary_figure_4_ddae019.pdf]

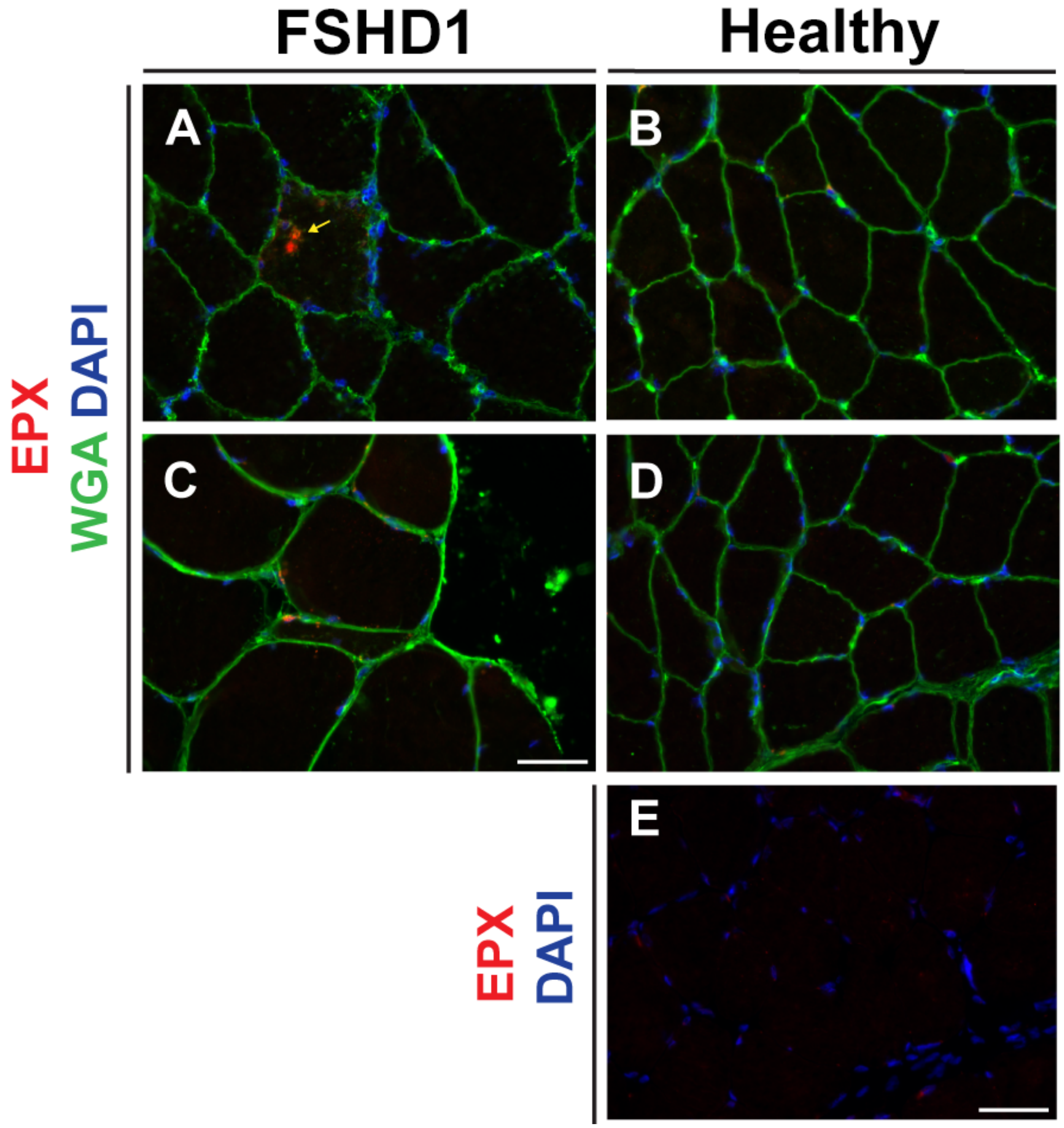

Figure S4- Eosinophil peroxidase expression in the skeletal muscle of human subject muscle biopsies. Immunofluorescence for eosinophil peroxidase (EPX), wheat germ agglutinin (WGA), and 4',6-diamidino-2-phenylindole (DAPI) in the human muscle biopsies from FSHD patients (A and C) and healthy (B, D and E) subjects. The yellow arrow indicates a nuclei close to the EPX positive staining. Scale bar: 50 µm.

Alt text: Full color immunostaining of muscle histological sections.
