# Supplementary material for: Muscle eosinophilia is a hallmark of chronic disease in facioscapulohumeral muscular dystrophy
Source: Hum Mol Genet. 2024 Feb 10;33(10):872–83. doi: 10.1093/hmg/ddae019 (PMC11070135; doi:10.1093/hmg/ddae019)
Supplement: Supplementary_Table_1_ddae019 [file supplementary_table_1_ddae019.pdf]

Cytokine levels (pg/mL)

Heat maps are colored so that green represents the lowest concentration, red the highest, and yellow the mean, ***per that analyte (column).***

Above highest limit of quantification
Below lowest limit of quantification
Failed

	Species	Sample	Sample Type	Homogenate Conc.	G-CSF	Eotaxin	GM-CSF	IFN-g	IL-1a	IL-1b	IL-2	IL-4	IL-3	IL-5	IL-6	IL-7	IL-9	IL-10
1	mouse	Animal FSHD #1	muscle homogenate	2000	<1.61↓	75	3.19	3.28	80.61	2.94	10.6	<4.66↓	<2.78↓	<3.82↓	4.19	<0.03↓	33.66	24.95
2	mouse	Animal FSHD #2	muscle homogenate	2000	<1.61↓	91.62	8.35	2.94	80.2	<0.02↓	10.3	<4.66↓	<2.78↓	<3.82↓	3.3	<0.03↓	<16.00↓	35.43
3	mouse	Animal FSHD #3	muscle homogenate	2000	<1.61↓	103.3	<1.01↓	3.03	77.74	3.43	11.3	<4.66↓	<2.78↓	<3.82↓	7.66	0.67	28.93	19.42
4	mouse	Animal FSHD #4	muscle homogenate	2000	<1.61↓	61.47	8.35	<1.75↓	44.09	1.44	7.07	<4.66↓	<2.78↓	14.76	<1.52↓	<0.03↓	38.14	3.69
5	mouse	Animal FSHD #5	muscle homogenate	2000	<1.61↓	56.69	11.67	3.45	56.76	<0.02↓	9.95	<4.66↓	<2.78↓	11.86	<1.52↓	<0.03↓	38.14	6.18
6	mouse	Animal FSHD #6	muscle homogenate	2000	<1.61↓	67.87	22.56	11.99	140.1	0.4	17.8	<4.66↓	<2.78↓	10.03	<1.52↓	1.39	46.5	24.18
7	mouse	Animal wild-type #7	muscle homogenate	2000	<1.61↓	15.34	<1.01↓	2.08	89.81	1.44	15.9	<4.66↓	<2.78↓	<3.82↓	7.13	5.45	<16.00↓	14.66
8	mouse	Animal wild-type #8	muscle homogenate	2000	<1.61↓	15.18	16.72	3.11	101.3	1.94	19.4	<4.66↓	<2.78↓	4.57	11.9	6.67	18.25	23.41
9	mouse	Animal wild-type #9	muscle homogenate	2000	<1.61↓	15.97	3.19	4.93	174.1	3.43	26.7	<4.66↓	<2.78↓	5.33	11.48	3.9	<16.00↓	51.89
10	mouse	Animal wild-type #10	muscle homogenate	2000	<1.61↓	15.89	11.67	1.99	101.3	0.93	17.3	<4.66↓	<2.78↓	9.71	7.92	2.29	<16.00↓	15.73
11	mouse	Animal wild-type #11	muscle homogenate	2000	<1.61↓	17.96	14.37	<1.75↓	105.4	2.44	16.4	<4.66↓	<2.78↓	<3.82↓	3.09	3.64	<16.00↓	13.58
12	mouse	Animal wild-type #12	muscle homogenate	2000	<1.61↓	33.32	15.58	<1.75↓	110.5	1.44	14.9	<4.66↓	<2.78↓	6.75	4.19	3.9	<16.00↓	27.41
13	mouse	Animal wild-type #13	muscle homogenate	2000	<1.61↓	12.86	14.37	<1.75↓	67.55	3.43	9.59	<4.66↓	<2.78↓	<3.82↓	<1.52↓	<0.03↓	<16.00↓	5.25
14	mouse	Animal wild-type #14	muscle homogenate	2000	<1.61↓	29.28	16.72	1.82	123	2.94	21.1	<4.66↓	<2.78↓	7.44	7.13	5.88	<16.00↓	26.49

	IL-12(p40)	IL-12(p70)	LIF	IL-13	LIX	IL-15	IL-17	IP-10	CXCL1	MCP-1	MIP-1a	MIP-1b	M-CSF	MIP-2	CXCL9	RANTES	VEGF	TNF-a
1	8.85	43.64	<0.25↓	<7.33↓	<8.57↓	<1.37↓	<2.40↓	21.43	4.2	<4.60↓	31.61	<4.78↓	<0.54↓	<16.00↓	18.87	<1.26↓	9.28	2.23
2	7.36	39.33	0.55	<7.33↓	<8.57↓	<1.37↓	<2.40↓	16.72	4.8	5.77	26.36	<4.78↓	1.49	<16.00↓	11.11	1.26	8.15	3.4
3	5.3	41.29	0.7	<7.33↓	<8.57↓	<1.37↓	<2.40↓	20.67	2.81	<4.60↓	23.42	<4.78↓	1.15	<16.00↓	12.36	<1.26↓	8.89	3.8
4	5.86	34.99	1.01	<7.33↓	<8.57↓	<1.37↓	<2.40↓	20.29	7.38	<4.60↓	23.42	<4.78↓	4.58	<16.00↓	50.64	1.27	16.38	2.9
5	7.17	32.23	0.41	<7.33↓	24.29	<1.37↓	<2.40↓	16.46	4.2	<4.60↓	32.02	<4.78↓	2.16	<16.00↓	26.37	<1.26↓	11.59	1.88
6	8.66	52.95	1.16	<7.33↓	88	<1.37↓	<2.40↓	17.74	7.66	5.77	36.67	<4.78↓	3.19	25.28	20.82	1.96	13.16	4.2
7	5.11	43.64	<0.25↓	<7.33↓	17.29	<1.37↓	<2.40↓	5.54	5.09	<4.60↓	31.61	<4.78↓	1.15	<16.00↓	<3.60↓	2.04	5.97	2.9
8	12.89	74.76	1.78	<7.33↓	45.29	16.21	<2.40↓	8.11	9.31	15.01	46.48	<4.78↓	5.29	<16.00↓	<3.60↓	3.22	9.62	5.8
9	9.03	50.64	0.26	<7.33↓	31.6	<1.37↓	<2.40↓	7.64	8.35	<4.60↓	35.55	<4.78↓	1.82	<16.00↓	<3.60↓	3.1	6.18	5.2
10	8.1	60.59	0.55	<7.33↓	13.15	<1.37↓	<2.40↓	6.29	5.96	<4.60↓	42.29	<4.78↓	<0.54↓	<16.00↓	<3.60↓	2.76	4	5.58
11	9.95	52.95	0.41	<7.33↓	22.77	<1.37↓	<2.40↓	6.63	9.58	7.76	48.94	<4.78↓	1.15	<16.00↓	<3.60↓	3.26	4.33	2.31
12	9.59	41.68	<0.25↓	<7.33↓	<8.57↓	<1.37↓	<2.40↓	6.5	7.93	<4.60↓	34.79	<4.78↓	4.58	<16.00↓	<3.60↓	1.69	5.37	4.82
13	10.69	56.79	<0.25↓	12.04	<8.57↓	<1.37↓	<2.40↓	4.02	15.47	<4.60↓	15.85	<4.78↓	1.15	<16.00↓	<3.60↓	1.39	3.32	3.23
14	9.59	46.37	0.48	17.27	47.91	2.92	<2.40↓	7.24	15.34	5.77	62.69	<4.78↓	2.5	<16.00↓	<3.60↓	3.85	6.79	4.67

Table S1- Cytokine/chemokine profile in the skeletal muscle from 3 month-old chronic FSHD-like mice. Luminex protein quantification of cytokines/chemokines in the skeletal muscle of 3 month-old chronic FSHD-like mice.
