# Supplementary material for: Muscle eosinophilia is a hallmark of chronic disease in facioscapulohumeral muscular dystrophy
Source: Hum Mol Genet. 2024 Feb 10;33(10):872–83. doi: 10.1093/hmg/ddae019 (PMC11070135; doi:10.1093/hmg/ddae019)
Supplement: Supplementary_Table_2_ddae019 [file supplementary_table_2_ddae019.pdf]

Cytokine levels (pg/mL)

Heat maps are colored so that green represents the lowest concentration, red the highest, and yellow the mean, ***per that analyte (column).***

Above highest limit of quantification
Below lowest limit of quantification
Failed

	Species	Sample	Gender	Sample Type	Homogenate Conc (ug/mL)	G-CSF	Eotaxin	GM-CSF	IFN-g	IL-1a	IL-1b	IL-2	IL-4	IL-3	IL-5	IL-6	IL-7	IL-9
1	mouse	Animal FSHD #1	Male	homogenate	2000	<1.78↓	76.03	<2.45↓	<2.58↓	685.46	<2.30↓	10.22	2.24	<1.79↓	<1.75↓	1.61	3.25	3.19
2	mouse	Animal FSHD #2	Male	homogenate	2000	<1.78↓	65.94	7.3	7.55	1359	4.15	23.22	<1.39↓	<1.79↓	<1.75↓	2.43	4.52	201.29
3	mouse	Animal FSHD #3	Male	homogenate	2000	<1.78↓	79.16	4.68	<2.58↓	1319	3.2	26.21	<1.39↓	<1.79↓	<1.75↓	1.93	4.08	322.46
4	mouse	Animal FSHD #4	Male	homogenate	2000	<1.78↓	21.17	<2.45↓	<2.58↓	695.53	2.96	11.35	<1.39↓	<1.79↓	<1.75↓	1.61	6.58	242.63
5	mouse	Animal FSHD #5	Male	homogenate	2000	<1.78↓	64.82	12.41	<2.58↓	938.42	4.15	16.37	<1.39↓	<1.79↓	<1.75↓	1.93	3.46	335.37
6	mouse	Animal FSHD #6	Female	homogenate	2000	<1.78↓	61.94	4.68	<2.58↓	905.8	3.2	15.99	<1.39↓	<1.79↓	<1.75↓	1.77	2.66	487.27
7	mouse	Animal FSHD #7	Female	homogenate	2000	<1.78↓	54.53	<2.45↓	2.83	1363	3.67	27	<1.39↓	<1.79↓	<1.75↓	2.26	4.3	335.37
8	mouse	Animal FSHD #8	Female	homogenate	2000	<1.78↓	62.77	<2.45↓	<2.58↓	955.61	2.72	14.65	<1.39↓	<1.79↓	<1.75↓	1.32	1.93	159.3
9	mouse	Animal FSHD #9	Female	homogenate	2000	<1.78↓	27.37	<2.45↓	<2.58↓	673.35	2.72	15.07	1.74	<1.79↓	<1.75↓	2.09	2.66	103.29
10	mouse	Animal wild-type #17	Male	homogenate	2000	<1.78↓	17.2	<2.45↓	<2.58↓	822.39	<2.30↓	15.16	<1.39↓	<1.79↓	<1.75↓	<0.87↓	3.46	159.3
11	mouse	Animal wild-type #18	Male	homogenate	2000	<1.78↓	17.73	<2.45↓	2.83	925.01	<2.30↓	19.88	<1.39↓	<1.79↓	<1.75↓	1.18	4.41	145.24
12	mouse	Animal wild-type #19	Male	homogenate	2000	<1.78↓	17.93	<2.45↓	<2.58↓	624.56	2.72	17.51	<1.39↓	<1.79↓	<1.75↓	1.46	4.3	131.18
13	mouse	Animal wild-type #20	Male	homogenate	2000	<1.78↓	18.19	<2.45↓	<2.58↓	612.27	<2.30↓	11.28	<1.39↓	<1.79↓	<1.75↓	1.39	3.46	131.18
14	mouse	Animal wild-type #21	Male	homogenate	2000	<1.78↓	13.99	<2.45↓	4.59	721.58	<2.30↓	15.29	<1.39↓	<1.79↓	<1.75↓	1.05	3.87	117.18
15	mouse	Animal wild-type #22	Male	homogenate	2000	<1.78↓	10.98	<2.45↓	2.83	512.53	<2.30↓	11.28	<1.39↓	<1.79↓	<1.75↓	1.25	3.25	145.24
16	mouse	Animal wild-type #10	Female	homogenate	2000	<1.78↓	50.28	<2.45↓	<2.58↓	855.53	2.72	12.39	<1.39↓	<1.79↓	<1.75↓	1.77	2.11	145.24
17	mouse	Animal wild-type #11	Female	homogenate	2000	<1.78↓	<3.73↓	<2.45↓	3.11	612.27	<2.30↓	14.84	<1.39↓	<1.79↓	<1.75↓	1.46	2.29	145.24
18	mouse	Animal wild-type #12	Female	homogenate	2000	<1.78↓	9.48	13.24	2.97	855.53	2.48	14.01	<1.39↓	<1.79↓	<1.75↓	1.18	4.08	296.3
19	mouse	Animal wild-type #13	Female	homogenate	2000	<1.78↓	12.79	2.94	<2.58↓	1103	<2.30↓	20.78	<1.39↓	<1.79↓	<1.75↓	1.54	4.3	360.83
20	mouse	Animal wild-type #14	Female	homogenate	2000	<1.78↓	13.99	<2.45↓	<2.58↓	982.26	<2.30↓	21.68	<1.39↓	<1.79↓	<1.75↓	1.77	3.56	215.16
21	mouse	Animal wild-type #15	Female	homogenate	2000	<1.78↓	8.86	<2.45↓	<2.58↓	1177	<2.30↓	24.93	<1.39↓	<1.79↓	<1.75↓	0.92	2.66	328.93
22	mouse	Animal wild-type #16	Female	homogenate	2000	<1.78↓	9.93	<2.45↓	<2.58↓	1046	<2.30↓	20.32	<1.39↓	<1.79↓	<1.75↓	<0.87↓	2.29	335.37

	IL-10	IL-12(p40)	IL-12(p70)	LIF	IL-13	LIX	IL-15	IL-17	IP-10	CXCL1	MCP-1	MIP-1a	MIP-1b	M-CSF	MIP-2	CXCL9	RANTES	VEGF	TNF-a
1	30.1	<1.33↓	549.55	<0.74↓	63.66	60.16	<4.44↓	3.89	8.98	30.67	15.19	48.31	<4.47↓	3.56	<6.64↓	20.98	<2.07↓	9.88	4.65
2	118.07	13.57	695.92	2.89	105.6	365.57	150.88	3.14	23.15	79.21	15.19	105.75	25.44	2.59	106.73	27.2	3.84	35.24	6.22
3	151.42	9.29	617.02	1.05	122.46	347.59	104.76	3.89	23.33	80.22	11.97	81.11	13.05	4.04	135.72	17.35	2.57	31.95	5.12
4	75.93	<1.33↓	541.14	1.42	102.64	72.12	29.47	3.01	2.51	30.76	8.85	38.68	15.82	2.59	121.69	8.33	<2.07↓	13.7	5.4
5	134.15	<1.33↓	585.36	1.29	74.37	148.17	24.09	2.56	27.67	38.54	25.25	45.81	23.2	4.28	149.03	37.4	<2.07↓	30.39	<3.22↓
6	110.63	<1.33↓	564.51	<0.74↓	89.19	137.69	48.26	<2.16↓	29.33	36.32	15.19	60.48	4.47	5.91	106.73	61.85	<2.07↓	24.07	<3.22↓
7	161.26	3.36	567.5	0.94	135.2	305.75	77.8	6.58	30.64	81.83	11.97	92.44	8.34	3.56	52.14	69.51	2.67	39.26	7.01
8	113.11	<1.33↓	449.87	<0.74↓	80.29	130.7	<4.44↓	3.8	14.87	36.81	8.85	75.05	<4.47↓	<0.97↓	<6.64↓	23.45	<2.07↓	26.82	6.13
9	141.56	<1.33↓	543.24	<0.74↓	123.98	239.01	104.76	2.29	10.7	51.64	8.85	67.02	<4.47↓	3.08	52.14	29.73	2.19	12.81	4.75
10	179.65	<1.33↓	515.78	<0.74↓	91.93	117.89	72.42	2.2	4.07	38.45	<2.26↓	49.13	<4.47↓	<0.97↓	25.73	3.63	<2.07↓	24.03	5.76
11	75.93	<1.33↓	578.23	<0.74↓	107.29	113.23	77.8	2.33	5.24	50.47	3.24	47.49	<4.47↓	<0.97↓	52.14	5.39	<2.07↓	21.07	8.04
12	66.68	<1.33↓	614.36	0.94	136.56	138.86	114.22	2.83	6.09	53.31	3.24	62.59	<4.47↓	<0.97↓	52.14	3.63	<2.07↓	17.61	8.29
13	98.23	<1.33↓	558.53	<0.74↓	74.37	77.28	21.39	<2.16↓	3.34	25.26	<2.26↓	39.61	<4.47↓	<0.97↓	52.14	3.63	<2.07↓	12.27	5.76
14	121.78	6.01	571.08	1.29	93.08	114.97	102.06	<2.16↓	4.62	51.81	5.91	44.96	<4.47↓	<0.97↓	52.14	3.63	<2.07↓	16.76	5.58
15	44.19	1.43	504.24	<0.74↓	57.97	71.54	13.27	<2.16↓	2.51	27.76	3.24	37.25	<4.47↓	<0.97↓	25.73	2.84	<2.07↓	15.56	7.7
16	56.27	<1.33↓	514.87	0.82	81.91	108.57	45.58	<2.16↓	11.05	32.63	3.24	46.23	11.57	3.56	52.14	28.47	<2.07↓	16.41	<3.22↓
17	552.47	1.61	569.29	<0.74↓	98.66	106.24	260.99	<2.16↓	6.42	77.52	<2.26↓	24.14	<4.47↓	<0.97↓	72.71	4.05	2.41	6.07	7.96
18	104.43	<1.33↓	538.13	<0.74↓	101.36	92.3	67.05	<2.16↓	4.22	39.2	15.19	32.73	29.71	6.37	185.66	4.48	<2.07↓	12.1	4.65
19	95.75	6.35	533.91	<0.74↓	159.17	146.42	112.87	2.29	5.67	55.82	5.91	47.49	10.01	2.09	135.72	7.32	<2.07↓	17.47	6.48
20	80.88	7.04	527.58	0.82	138.46	117.89	91.27	<2.16↓	5.19	59.42	5.91	44.1	4.47	1.06	106.73	4.93	<2.07↓	20.78	7.27
21	139.09	2.41	558.83	<0.74↓	140.36	122.55	37.53	<2.16↓	12.68	64.87	<2.26↓	48.31	<4.47↓	1.58	72.71	5.85	<2.07↓	20.41	6.39
22	107.53	<1.33↓	496.93	<0.74↓	124.53	134.78	44.24	2.2	8.02	52.39	<2.26↓	53.08	<4.47↓	<0.97↓	52.14	3.63	<2.07↓	22.17	5.21

Table S2- Cytokine/chemokine profile in the skeletal muscle from 6 month-old chronic FSHD-like mice. Luminex protein quantification of cytokines/chemokines in the skeletal muscle of 6 month-old chronic FSHD-like mice.
