# Supplementary material for: Muscle eosinophilia is a hallmark of chronic disease in facioscapulohumeral muscular dystrophy
Source: Hum Mol Genet. 2024 Feb 10;33(10):872–83. doi: 10.1093/hmg/ddae019 (PMC11070135; doi:10.1093/hmg/ddae019)
Supplement: Supplementary_Table_3_ddae019 [file supplementary_table_3_ddae019.pdf]

Cytokine levels (pg/mL)

Heat maps are colored so that green represents the lowest concentration, red the highest, and yellow the mean, ***per that analyte (column).***

Above highest limit of quantification
Below lowest limit of quantification
Failed

	Species	Sample	Sample Type	Homogenate Conc (ug/mL)	G-CSF	Eotaxin	GM-CSF	IFN-g	IL-1a	IL-1b	IL-2	IL-4	IL-3	IL-5	IL-6	IL-7	IL-9	IL-10	IL-12(p40)	IL-12(p70)
1	mouse	Animal FSHD #1	muscle homogenate	2000	<0.05↓	56.45	<2.08↓	<2.49↓	122.1	4.23	3.14	<2.02↓	<0.02↓	<2.06↓	2.47	6.46	65.27	4.35	<0.40↓	<16.00↓
2	mouse	Animal FSHD #2	muscle homogenate	2000	<0.05↓	76.02	<2.08↓	<2.49↓	116.1	4.99	3.72	<2.02↓	<0.02↓	<2.06↓	4.41	6.25	39.11	9.44	<0.40↓	<16.00↓
3	mouse	Animal FSHD #3	muscle homogenate	2000	<0.05↓	152.14	<2.08↓	<2.49↓	254.2	5.38	7.88	<2.02↓	<0.02↓	<2.06↓	5.09	8.26	68.49	8.68	<0.40↓	<16.00↓
4	mouse	Animal FSHD #4	muscle homogenate	2000	<0.05↓	98.41	<2.08↓	3.85	336.6	5.76	8.5	<2.02↓	<0.02↓	<2.06↓	6.66	6.98	58.79	17.34	<0.40↓	<16.00↓
5	mouse	Animal FSHD #5	muscle homogenate	2000	<0.05↓	17.83	<2.08↓	<2.49↓	204.5	3.47	6.02	<2.02↓	<0.02↓	<2.06↓	<2.37↓	6.67	55.53	7.19	<0.40↓	<16.00↓
6	mouse	Animal FSHD #6	muscle homogenate	2000	<0.05↓	110.67	<2.08↓	18.05	511.5	5.18	7.96	<2.02↓	<0.02↓	<2.06↓	8.45	6.15	58.79	9.06	<0.40↓	<16.00↓
7	mouse	Animal FSHD #7	muscle homogenate	2000	<0.05↓	158.29	<2.08↓	7.48	362.5	5.76	6.1	<2.02↓	<0.02↓	<2.06↓	8.27	6.56	65.27	14.14	<0.40↓	<16.00↓
8	mouse	Animal FSHD #8	muscle homogenate	2000	<0.05↓	106.79	<2.08↓	8.06	332.2	5.38	6.02	<2.02↓	<0.02↓	<2.06↓	6.66	6.15	78.09	5.74	<0.40↓	<16.00↓
9	mouse	Animal FSHD #9	muscle homogenate	2000	<0.05↓	65.64	<2.08↓	<2.49↓	199.4	4.61	5.25	<2.02↓	0.2	<2.06↓	<2.37↓	6.77	71.71	<2.96↓	<0.40↓	<16.00↓
10	mouse	Animal wild-type #10	muscle homogenate	2000	<0.05↓	21.75	<2.08↓	<2.49↓	526.7	3.47	4.09	<2.02↓	<0.02↓	8.56	<2.37↓	7.4	78.09	7.93	<0.40↓	<16.00↓
11	mouse	Animal wild-type #11	muscle homogenate	2000	<0.05↓	13.16	<2.08↓	<2.49↓	287.2	3.09	5.08	<2.02↓	<0.02↓	9.67	<2.37↓	5.94	39.11	3.36	<0.40↓	<16.00↓
12	mouse	Animal wild-type #12	muscle homogenate	2000	<0.05↓	25.8	<2.08↓	<2.49↓	338.8	5.38	15.69	<2.02↓	<0.02↓	<2.06↓	5.09	7.4	138.9	20.58	<0.40↓	<16.00↓
13	mouse	Animal wild-type #13	muscle homogenate	2000	<0.05↓	22.95	<2.08↓	<2.49↓	332.2	6.54	5.51	<2.02↓	<0.02↓	<2.06↓	3.58	6.98	45.7	12.55	<0.40↓	<16.00↓
14	mouse	Animal wild-type #14	muscle homogenate	2000	<0.05↓	25.18	<2.08↓	<2.49↓	239.7	5.38	5.25	<2.02↓	<0.02↓	<2.06↓	3.1	6.77	45.7	9.44	<0.40↓	<16.00↓
15	mouse	Animal wild-type #15	muscle homogenate	2000	<0.05↓	24.13	<2.08↓	<2.49↓	199.4	4.61	2.84	<2.02↓	<0.02↓	<2.06↓	2.47	6.15	58.79	<2.96↓	<0.40↓	<16.00↓
16	mouse	Animal wild-type #16	muscle homogenate	2000	<0.05↓	33.71	38.48	13.98	207.1	5.76	2.94	2.07	<0.02↓	3.66	3.02	6.98	71.71	9.44	10.15	<16.00↓
17	mouse	Animal wild-type #17	muscle homogenate	2000	<0.05↓	33.47	<2.08↓	<2.49↓	214.7	4.99	7.25	<2.02↓	<0.02↓	<2.06↓	<2.37↓	6.25	71.71	7.19	<0.40↓	<16.00↓

	Sample	LIF	IL-13	LIX	IL-15	IL-17	IP-10	CXCL1	MCP-1	MIP-1a	MIP-1b	M-CSF	MIP-2	CXCL9	RANTES	VEGF	TNF-a
1	Animal FSHD #1	0.49	<5.90↓	<1.98↓	<2.32↓	<2.83↓	25.81	3.79	<16.00↓	90.85	<13.67↓	<1.50↓	<2.96↓	30.28	4.42	25.08	<3.07↓
2	Animal FSHD #2	1.18	<5.90↓	<1.98↓	<2.32↓	3.07	18.92	<2.35↓	<16.00↓	77.72	<13.67↓	<1.50↓	<2.96↓	16.77	1.92	21.26	<3.07↓
3	Animal FSHD #3	<0.06↓	<5.90↓	<1.98↓	<2.32↓	4.44	14.67	<2.35↓	<16.00↓	67.63	30.92	1.66	<2.96↓	12.64	4.12	18.17	<3.07↓
4	Animal FSHD #4	0.49	<5.90↓	<1.98↓	<2.32↓	4.23	18.16	<2.35↓	<16.00↓	70.07	<13.67↓	1.66	<2.96↓	8.39	4.92	22.9	<3.07↓
5	Animal FSHD #5	0.16	<5.90↓	<1.98↓	<2.32↓	<2.83↓	<4.16↓	<2.35↓	<16.00↓	64.55	<13.67↓	<1.50↓	<2.96↓	<2.81↓	<1.86↓	15.79	<3.07↓
6	Animal FSHD #6	0.55	<5.90↓	<1.98↓	<2.32↓	3.13	18.27	<2.35↓	<16.00↓	64.55	<13.67↓	<1.50↓	<2.96↓	12.42	<1.86↓	13.94	<3.07↓
7	Animal FSHD #7	<0.06↓	<5.90↓	<1.98↓	<2.32↓	<2.83↓	31.06	<2.35↓	<16.00↓	66.62	<13.67↓	<1.50↓	<2.96↓	19.73	<1.86↓	35.65	<3.07↓
8	Animal FSHD #8	0.29	<5.90↓	<1.98↓	6.08	<2.83↓	15.7	<2.35↓	<16.00↓	67.13	<13.67↓	<1.50↓	<2.96↓	7.53	<1.86↓	22.42	<3.07↓
9	Animal FSHD #9	<0.06↓	<5.90↓	<1.98↓	6.08	2.97	9.25	<2.35↓	<16.00↓	68.62	<13.67↓	<1.50↓	<2.96↓	5.85	<1.86↓	18.92	<3.07↓
10	Animal wild-type #10	<0.06↓	<5.90↓	<1.98↓	<2.32↓	4.28	<4.16↓	10.38	<16.00↓	66.62	<13.67↓	<1.50↓	<2.96↓	<2.81↓	<1.86↓	14.07	<3.07↓
11	Animal wild-type #11	<0.06↓	6.96	<1.98↓	<2.32↓	<2.83↓	<4.16↓	5.05	<16.00↓	45.59	<13.67↓	<1.50↓	16.43	<2.81↓	2.95	13.36	<3.07↓
12	Animal wild-type #12	<0.06↓	7.53	110.7	<2.32↓	<2.83↓	<4.16↓	<2.35↓	<16.00↓	90.85	<13.67↓	<1.50↓	<2.96↓	<2.81↓	5.71	12.3	<3.07↓
13	Animal wild-type #13	<0.06↓	<5.90↓	<1.98↓	<2.32↓	<2.83↓	<4.16↓	<2.35↓	<16.00↓	74.24	<13.67↓	2.29	<2.96↓	<2.81↓	<1.86↓	13.56	<3.07↓
14	Animal wild-type #14	<0.06↓	<5.90↓	<1.98↓	<2.32↓	<2.83↓	<4.16↓	<2.35↓	<16.00↓	56.62	<13.67↓	<1.50↓	<2.96↓	<2.81↓	<1.86↓	4.53	<3.07↓
15	Animal wild-type #15	<0.06↓	<5.90↓	<1.98↓	<2.32↓	<2.83↓	<4.16↓	<2.35↓	<16.00↓	50.06	<13.67↓	<1.50↓	<2.96↓	<2.81↓	<1.86↓	14.52	<3.07↓
16	Animal wild-type #16	0.55	<5.90↓	<1.98↓	60.62	<2.83↓	5.74	<2.35↓	<16.00↓	51.45	42.96	4.91	398.31	<2.81↓	<1.86↓	16.04	<3.07↓
17	Animal wild-type #17	<0.06↓	<5.90↓	<1.98↓	<2.32↓	<2.83↓	<4.16↓	<2.35↓	<16.00↓	61.3	<13.67↓	<1.50↓	<2.96↓	<2.81↓	<1.86↓	21.81	<3.07↓

Table S3- Cytokine/chemokine profile in the skeletal muscle from 14-18 month-old chronic FSHD-like mice. Luminex protein quantification of cytokines/chemokines in the skeletal muscle of 14-18 month-old chronic FSHD-like mice.
