# Supplementary material for: Muscle eosinophilia is a hallmark of chronic disease in facioscapulohumeral muscular dystrophy
Source: Hum Mol Genet. 2024 Feb 10;33(10):872–83. doi: 10.1093/hmg/ddae019 (PMC11070135; doi:10.1093/hmg/ddae019)
Supplement: Supplementary_Table_4_ddae019 [file supplementary_table_4_ddae019.pdf]

Cytokine levels (pg/mL)

Heat maps are colored so that green represents the lowest concentration, red the highest, and yellow the mean, ***per that analyte (column).***

Above highest limit of quantification
Below lowest limit of quantification
Failed

	Species	Sample	Sample Type	G-CSF	Eotaxin	GM-CSF	IFN-g	IL-1a	IL-1b	IL-2	IL-4
1	mouse	Animal FSHD #1	serum	115.37	900.21	20.35	<2.84↓	4.7	<2.71↓	<2.66↓	<0.88↓
2	mouse	Animal FSHD #2	serum	159.06	653.63	<3.04↓	<2.84↓	10.68	<2.71↓	<2.66↓	<0.88↓
3	mouse	Animal FSHD #3	serum	133.71	633.24	<3.04↓	<2.84↓	10.68	<2.71↓	<2.66↓	<0.88↓
4	mouse	Animal FSHD #4	serum	167.75	1070	<3.04↓	<2.84↓	84.14	<2.71↓	<2.66↓	<0.88↓
5	mouse	Animal FSHD #5	serum	50.89	1089	<3.04↓	<2.84↓	208.1	<2.71↓	<2.66↓	<0.88↓
6	mouse	Animal FSHD #6	serum	267.43	1495	<3.04↓	<2.84↓	105	<2.71↓	<2.66↓	<0.88↓
7	mouse	Animal wild-type #7	serum	95.28	659.82	15.3	<2.84↓	42.52	<2.71↓	<2.66↓	<0.88↓
8	mouse	Animal wild-type #8	serum	99.71	541.37	5.44	<2.84↓	98.28	37.76	<2.66↓	<0.88↓
9	mouse	Animal wild-type #9	serum	163.8	446.98	9.2	<2.84↓	16.48	<2.71↓	<2.66↓	<0.88↓
10	mouse	Animal wild-type #10	serum	226.69	465.64	5.44	<2.84↓	4.7	<2.71↓	<2.66↓	<0.88↓
11	mouse	Animal wild-type #11	serum	131.72	656.69	<3.04↓	<2.84↓	16.48	<2.71↓	<2.66↓	<0.88↓
12	mouse	Animal wild-type #12	serum	66.72	598.57	<3.04↓	<2.84↓	4.7	<2.71↓	<2.66↓	<0.88↓
13	mouse	Animal wild-type #13	serum	79.85	609.03	<3.04↓	<2.84↓	32.65	<2.71↓	<2.66↓	<0.88↓
14	mouse	Animal wild-type #14	serum	53.41	448.59	<3.04↓	<2.84↓	10.68	<2.71↓	<2.66↓	<0.88↓

	IL-3	IL-5	IL-6	IL-7	IL-9	IL-10	IL-12(p40)	IL-12(p70)	LIF	IL-13	LIX	IL-15
1	<1.23↓	<2.85↓	<2.89↓	<2.03↓	98.83	<2.83↓	<2.17↓	<4.71↓	<2.50↓	<7.25↓	464.1	<3.07↓
2	<1.23↓	4.71	<2.89↓	<2.03↓	103.3	<2.83↓	<2.17↓	<4.71↓	<2.50↓	10	231.3	<3.07↓
3	<1.23↓	6.97	<2.89↓	<2.03↓	22.01	<2.83↓	<2.17↓	<4.71↓	<2.50↓	<7.25↓	456.3	<3.07↓
4	<1.23↓	5.27	<2.89↓	<2.03↓	91.89	<2.83↓	3.35	<4.71↓	<2.50↓	<7.25↓	951.9	<3.07↓
5	<1.23↓	<2.85↓	<2.89↓	<2.03↓	22.01	<2.83↓	5.77	<4.71↓	<2.50↓	<7.25↓	968.1	52.77
6	<1.23↓	<2.85↓	<2.89↓	<2.03↓	N/A	<2.83↓	3.35	<4.71↓	<2.50↓	26.06	889.8	<3.07↓
7	<1.23↓	<2.85↓	<2.89↓	<2.03↓	<4.25↓	<2.83↓	5.77	<4.71↓	<2.50↓	<7.25↓	496.6	<3.07↓
8	3.83	<2.85↓	<2.89↓	<2.03↓	49.22	<2.83↓	<2.17↓	<4.71↓	<2.50↓	<7.25↓	626	<3.07↓
9	<1.23↓	<2.85↓	<2.89↓	<2.03↓	127.7	<2.83↓	<2.17↓	<4.71↓	<2.50↓	<7.25↓	220.6	<3.07↓
10	<1.23↓	<2.85↓	9.96	<2.03↓	62.51	<2.83↓	3.35	<4.71↓	<2.50↓	<7.25↓	177.7	<3.07↓
11	<1.23↓	8.11	<2.89↓	<2.03↓	103.3	<2.83↓	<2.17↓	<4.71↓	<2.50↓	<7.25↓	92.15	<3.07↓
12	<1.23↓	10.09	<2.89↓	<2.03↓	65.54	<2.83↓	<2.17↓	<4.71↓	<2.50↓	<7.25↓	<2.75↓	<3.07↓
13	<1.23↓	4.3	<2.89↓	<2.03↓	74.1	<2.83↓	<2.17↓	<4.71↓	<2.50↓	<7.25↓	538.9	<3.07↓
14	<1.23↓	4.99	<2.89↓	<2.03↓	41.58	<2.83↓	<2.17↓	<4.71↓	<2.50↓	<7.25↓	424.3	<3.07↓

	IL-17	IP-10	CXCL1	MCP-1	MIP-1a	MIP-1b	M-CSF	MIP-2	CXCL9	RANTES	VEGF	TNF-a
1	<0.88↓	84.64	74.29	3.16	<3.14↓	<4.62↓	6.13	<2.79↓	66.04	9.42	<2.26↓	<3.00↓
2	<0.88↓	78.98	135.31	3.16	3.21	12.05	<2.74↓	<2.79↓	63.22	12.06	<2.26↓	<3.00↓
3	<0.88↓	59.77	63.03	<2.63↓	10.13	<4.62↓	<2.74↓	<2.79↓	53.27	4.53	<2.26↓	<3.00↓
4	1.84	103.7	217.89	3.16	<3.14↓	<4.62↓	6.13	<2.79↓	98.91	16.64	<2.26↓	<3.00↓
5	1.19	70.66	146.75	16.24	<3.14↓	<4.62↓	<2.74↓	<2.79↓	96.22	16.64	<2.26↓	<3.00↓
6	1.69	83.43	137.39	13	33.47	25.59	<2.74↓	12.74	119.51	10.78	<2.26↓	<3.00↓
7	<0.88↓	74.53	17.98	25.58	10.13	41.32	8.02	29.55	73.89	10.78	<2.26↓	<3.00↓
8	<0.88↓	69.69	110.33	45.17	<3.14↓	25.59	<2.74↓	<2.79↓	63.87	18.21	<2.26↓	14.72
9	<0.88↓	67.83	76.32	9.71	10.13	34.13	<2.74↓	<2.79↓	61.22	13.27	<2.26↓	4.28
10	<0.88↓	67.13	75.74	9.71	10.13	32.16	<2.74↓	<2.79↓	45.62	9.42	<2.26↓	<3.00↓
11	<0.88↓	78.01	89.26	13	10.13	30.09	<2.74↓	<2.79↓	81.81	15.01	<2.26↓	<3.00↓
12	<0.88↓	65.76	30.08	<2.63↓	27.06	14.01	<2.74↓	<2.79↓	68.58	<4.27↓	<2.26↓	<3.00↓
13	<0.88↓	56.78	15.93	<2.63↓	<3.14↓	<4.62↓	<2.74↓	<2.79↓	44.55	<4.27↓	<2.26↓	<3.00↓
14	<0.88↓	66.89	39.86	<2.63↓	<3.14↓	<4.62↓	<2.74↓	<2.79↓	65.39	7.57	<2.26↓	6.04

Table S4- Cytokine/chemokine profile in the serum from 3 month-old chronic FSHD-like mice. Luminex protein quantification of cytokines/chemokines in the serum of 3 month-old chronic FSHD-like mice.
