# Supplementary material for: Muscle eosinophilia is a hallmark of chronic disease in facioscapulohumeral muscular dystrophy
Source: Hum Mol Genet. 2024 Feb 10;33(10):872–83. doi: 10.1093/hmg/ddae019 (PMC11070135; doi:10.1093/hmg/ddae019)
Supplement: Supplementary_Table_5_ddae019 [file supplementary_table_5_ddae019.pdf]

Cytokine levels (pg/mL)

Heat maps are colored so that green represents the lowest concentration, red the highest, and yellow the mean, ***per that analyte (column).***

Above highest limit of quantification
Below lowest limit of quantification
Failed

	Species	Sample	Sample Type	G-CSF	Eotaxin	GM-CSF	IFN-g	IL-1a	IL-1b	IL-2	IL-4	IL-3	IL-5	IL-6	IL-7	IL-9	IL-10
1	mouse	Animal FSHD #1	serum	155.08	1178	29.17	6.34	83.76	6.36	7.82	4.01	<0.79↓	3.97	5.64	5.85	252.05	4.58
2	mouse	Animal FSHD #2	serum	177.74	775.02	<2.14↓	<0.61↓	32.83	1.11	<0.42↓	<1.29↓	<0.79↓	4.27	<2.45↓	14.95	134.12	1.17
3	mouse	Animal FSHD #3	serum	322.63	1007	11	<0.61↓	285.97	2.73	<0.42↓	<1.29↓	<0.79↓	4.57	<2.45↓	<0.49↓	27.08	<0.30↓
4	mouse	Animal FSHD #4	serum	242.58	1211	5.72	<0.61↓	129.32	<0.95↓	<0.42↓	<1.29↓	<0.79↓	4.57	<2.45↓	<0.49↓	126.14	1.49
5	mouse	Animal FSHD #5	serum	402.55	953.98	8.34	<0.61↓	206.96	1.11	<0.42↓	<1.29↓	<0.79↓	0.66	<2.45↓	24.72	83.85	<0.30↓
6	mouse	Animal FSHD #6	serum	158.17	1558	<2.14↓	<0.61↓	22.03	1.11	<0.42↓	<1.29↓	<0.79↓	4.42	<2.45↓	<0.49↓	186.28	<0.30↓
7	mouse	Animal FSHD #7	serum	265.47	950.77	18.95	2.35	58.32	<0.95↓	<0.42↓	<1.29↓	<0.79↓	9.1	<2.45↓	<0.49↓	391.14	<0.30↓
8	mouse	Animal FSHD #8	serum	263.39	1385	8.34	<0.61↓	46.5	1.11	3.99	<1.29↓	<0.79↓	7.73	<2.45↓	<0.49↓	230	<0.30↓
9	mouse	Animal FSHD #9	serum	167.43	1441	<2.14↓	1.65	362.46	3.2	6.71	4.49	1.43	10.98	<2.45↓	19.8	193.26	0.85
10	mouse	Animal FSHD #10	serum	142.07	1123	38.82	47.08	353.09	31.06	0.94	3.17	7.13	13.86	14.68	<0.49↓	141.96	9.98
11	mouse	Animal wild-type #11	serum	270.33	1077	<2.14↓	1.65	19.71	1.46	<0.42↓	<1.29↓	<0.79↓	15.65	<2.45↓	<0.49↓	96.99	<0.30↓
12	mouse	Animal wild-type #12	serum	204.93	1259	<2.14↓	<0.61↓	72.65	<0.95↓	<0.42↓	<1.29↓	<0.79↓	8.01	<2.45↓	19.65	36.98	1.49
13	mouse	Animal wild-type #13	serum	477.76	982.39	<2.14↓	3.1	59.05	1.11	0.73	<1.29↓	<0.79↓	18.17	<2.45↓	0.7	189.78	4.58
14	mouse	Animal wild-type #14	serum	363	3171	3.19	<0.61↓	308.13	1.11	<0.42↓	<1.29↓	<0.79↓	6.89	<2.45↓	<0.49↓	105.52	2.12
15	mouse	Animal wild-type #15	serum	283.89	1381	3.19	<0.61↓	85.13	1.46	<0.42↓	<1.29↓	<0.79↓	14.89	<2.45↓	0.95	88.27	<0.30↓
16	mouse	Animal wild-type #16	serum	125.65	977.12	17.64	3.49	25.11	<0.95↓	11.01	<1.29↓	<0.79↓	22.35	<2.45↓	6.14	210.3	1.49
17	mouse	Animal wild-type #17	serum	122.24	1288	24.13	3.39	312.51	12.28	21.78	30.85	3.61	5.46	<2.45↓	21.02	74.86	2.74
18	mouse	Animal wild-type #18	serum	221.15	1105	<2.14↓	1.16	11.39	1.46	2.57	<1.29↓	1.23	3.36	<2.45↓	<0.49↓	65.67	0.85
19	mouse	Animal wild-type #19	serum	140.7	1176	8.34	0.86	258.71	3.2	0.52	1.72	<0.79↓	12.3	<2.45↓	<0.49↓	70.29	1.49
20	mouse	Animal wild-type #20	serum	197.69	1087	<2.14↓	<0.61↓	337.97	1.11	<0.42↓	<1.29↓	<0.79↓	3.97	<2.45↓	<0.49↓	65.67	<0.30↓
21	mouse	Animal wild-type #21	serum	190.81	647.86	3.19	<0.61↓	52.45	1.11	<0.42↓	<1.29↓	<0.79↓	5.46	<2.45↓	<0.49↓	70.29	<0.30↓
22	mouse	Animal wild-type #22	serum	321.24	922.06	3.19	1.01	82.39	<0.95↓	<0.42↓	<1.29↓	<0.79↓	5.16	<2.45↓	10.19	17.12	0.85

	IL-12(p40)	IL-12(p70)	LIF	IL-13	LIX	IL-15	IL-17	IP-10	CXCL1	MCP-1	MIP-1a	MIP-1b	M-CSF	MIP-2	CXCL9	RANTES	VEGF	TNF-a
1	11.8	6.34	<1.82↓	127.97	<1.88↓	30.2	2.7	173.41	302.09	26.77	75.14	12.52	7.12	70.08	259.33	2.44	10.22	6.34
2	9.94	<1.25↓	<1.82↓	48.97	1246	2.06	1.83	181.49	177.68	20.92	<1.37↓	11.52	<3.16↓	32.97	312.31	4.11	0.76	6.34
3	5.66	<1.25↓	<1.82↓	57.24	1500	4.56	<1.40↓	178.58	301.29	9.05	5.55	33.3	<3.16↓	70.08	231.86	3.6	0.72	<2.98↓
4	2.47	<1.25↓	<1.82↓	24.94	558.45	<1.33↓	<1.40↓	154.14	85.01	14.22	<1.37↓	21.12	4.35	32.97	102.34	2.46	<0.68↓	<2.98↓
5	11.33	<1.25↓	18.62	54.48	1281	448.99	1.83	166.08	326.96	16.09	<1.37↓	22.95	<3.16↓	104.84	375.75	3.7	0.83	<2.98↓
6	2.47	<1.25↓	<1.82↓	30.12	1075	230.59	<1.40↓	163.54	222.23	27.49	<1.37↓	18.33	<3.16↓	<2.95↓	638.64	5.5	<0.68↓	<2.98↓
7	10.86	<1.25↓	<1.82↓	68.33	<1.88↓	7.91	<1.40↓	193.19	231.84	14.97	<1.37↓	33.3	<3.16↓	15.36	895.82	4.64	0.69	<2.98↓
8	<2.36↓	<1.25↓	<1.82↓	38.1	1040	7.91	<1.40↓	173.41	234.27	13.48	<1.37↓	<2.77↓	<3.16↓	<2.95↓	551.86	2.28	<0.68↓	<2.98↓
9	8.16	4.53	1.83	57.24	294.85	79.75	1.53	190.85	206.51	28.93	40.32	19.27	<3.16↓	79.85	596.37	3.57	0.76	8.35
10	14.21	33.75	<1.82↓	65.55	1659	33.17	3.68	168.11	223.95	61.47	<1.37↓	41.27	4.92	59.28	455.62	9.06	<0.68↓	43.27
11	9.94	<1.25↓	<1.82↓	43.51	1388	16.61	<1.40↓	142.96	74.55	10.51	2.42	3.19	<3.16↓	<2.95↓	214.39	2.79	1.29	<2.98↓
12	7.51	<1.25↓	<1.82↓	24.94	1025	4.56	<1.40↓	155.73	20.48	11.99	<1.37↓	22.95	<3.16↓	32.97	265.39	4.22	0.83	<2.98↓
13	10.4	<1.25↓	<1.82↓	31.44	220.09	55.59	<1.40↓	254.38	192.39	11.25	8.61	13.51	14.37	163.64	276.46	<1.64↓	1.53	<2.98↓
14	14.7	<1.25↓	<1.82↓	82.24	2057	21.75	<1.40↓	159.99	112.25	26.77	2.42	46.54	<3.16↓	97.1	330.04	7.29	0.9	4.83
15	10.86	<1.25↓	<1.82↓	47.6	454	122.79	<1.40↓	138.64	109.91	43.48	<1.37↓	21.12	<3.16↓	47.11	222.63	5.94	<0.68↓	<2.98↓
16	9.94	1.62	1.97	55.86	<1.88↓	180.66	<1.40↓	178.87	189.44	22.39	94.38	6.39	<3.16↓	<2.95↓	543.63	2.82	5.32	3.33
17	19.76	23.72	2.05	76.68	2071	11.96	<1.40↓	230.57	224.26	77.01	259.86	109.99	18.34	177.27	299.43	78.6	7.99	28.33
18	<2.36↓	3.44	<1.82↓	80.85	811.61	27.3	<1.40↓	163.11	246.03	16.83	<1.37↓	28.24	<3.16↓	32.97	241.06	4.42	<0.68↓	3.33
19	9.94	2.02	<1.82↓	19.91	2161	<1.33↓	<1.40↓	153.85	83.03	9.78	5.55	7.44	9.77	153.78	166.38	5.17	0.69	<2.98↓
20	<2.36↓	1.62	<1.82↓	27.52	1416	3.2	<1.40↓	138.78	177.37	<8.50↓	<1.37↓	8.47	3.78	137.52	98.03	2.68	0.69	<2.98↓
21	6.67	1.25	<1.82↓	30.12	753.9	4.56	<1.40↓	132.74	169.03	<8.50↓	<1.37↓	11.52	<3.16↓	47.11	51	2.7	0.69	<2.98↓
22	<2.36↓	<1.25↓	<1.82↓	30.12	858.98	3.2	<1.40↓	143.25	127.61	<8.50↓	<1.37↓	39.72	<3.16↓	<2.95↓	263.38	6.73	0.76	<2.98↓

Table S5- Cytokine/chemokine profile in the serum from 6 month-old chronic FSHD-like mice. Luminex protein quantification of cytokines/chemokines in the serum of 6 month-old chronic FSHD-like mice.
