# Supplementary material for: Muscle eosinophilia is a hallmark of chronic disease in facioscapulohumeral muscular dystrophy
Source: Hum Mol Genet. 2024 Feb 10;33(10):872–83. doi: 10.1093/hmg/ddae019 (PMC11070135; doi:10.1093/hmg/ddae019)
Supplement: Supplementary_Table_6_ddae019 [file supplementary_table_6_ddae019.pdf]

Cytokine levels (pg/mL)

Heat maps are colored so that green represents the lowest concentration, red the highest, and yellow the mean, ***per that analyte (column).***

Above highest limit of quantification
Below lowest limit of quantification
Failed

	Species	Sample	Sample Type	G-CSF	Eotaxin	GM-CSF	IFN-g	IL-1a	IL-1b	IL-2	IL-4	IL-3	IL-5	IL-6	IL-7	IL-9	IL-10
1	mouse	Animal FSHD #1	serum	348.99	437.86	<3.04↓	<2.84↓	<2.88↓	<2.71↓	<2.66↓	<0.88↓	<1.23↓	<2.85↓	26.03	3.78	62.51	8.75
2	mouse	Animal FSHD #2	serum	294.07	553.24	13.89	<2.84↓	4.7	<2.71↓	<2.66↓	<0.88↓	<1.23↓	4.99	4.02	<2.03↓	49.22	<2.83↓
3	mouse	Animal FSHD #3	serum	105.75	518.62	<3.04↓	<2.84↓	4.7	<2.71↓	<2.66↓	<0.88↓	<1.23↓	4.16	<2.89↓	2.12	94.24	<2.83↓
4	mouse	Animal FSHD #4	serum	259.99	778.19	<3.04↓	<2.84↓	129.6	<2.71↓	<2.66↓	<0.88↓	<1.23↓	4.71	12.6	5.68	87.06	<2.83↓
5	mouse	Animal FSHD #5	serum	164.99	1227	5.44	<2.84↓	<2.88↓	<2.71↓	<2.66↓	<0.88↓	<1.23↓	13.68	<2.89↓	41.22	89.49	<2.83↓
6	mouse	Animal FSHD #6	serum	105.35	453.24	<3.04↓	7.03	27.46	8.1	<2.66↓	<0.88↓	<1.23↓	<2.85↓	9.22	<2.03↓	62.51	<2.83↓
7	mouse	Animal FSHD #7	serum	113.77	493.13	9.2	<2.84↓	190.6	<2.71↓	4.88	<0.88↓	1.79	4.16	<2.89↓	<2.03↓	52.76	<2.83↓
8	mouse	Animal FSHD #8	serum	68.37	825.31	<3.04↓	<2.84↓	68.75	<2.71↓	<2.66↓	<0.88↓	<1.23↓	<2.85↓	<2.89↓	9.94	123.9	<2.83↓
9	mouse	Animal FSHD #9	serum	105.75	598.07	<3.04↓	<2.84↓	10.68	<2.71↓	2.82	<0.88↓	<1.23↓	<2.85↓	<2.89↓	<2.03↓	82.04	<2.83↓
10	mouse	Animal wild-type #10	serum	138.08	764.92	<3.04↓	<2.84↓	<2.88↓	<2.71↓	<2.66↓	<0.88↓	<1.23↓	6.97	<2.89↓	<2.03↓	22.01	<2.83↓
11	mouse	Animal wild-type #11	serum	224.33	679.77	<3.04↓	<2.84↓	7.71	<2.71↓	<2.66↓	<0.88↓	<1.23↓	34.48	49.43	42.85	62.51	<2.83↓
12	mouse	Animal wild-type #12	serum	85.96	885.14	<3.04↓	<2.84↓	10.68	<2.71↓	5.84	<0.88↓	<1.23↓	7.54	<2.89↓	<2.03↓	49.22	<2.83↓
13	mouse	Animal wild-type #13	serum	52.57	758.57	<3.04↓	<2.84↓	<2.88↓	<2.71↓	3.38	<0.88↓	<1.23↓	4.57	<2.89↓	8.47	45.51	<2.83↓
14	mouse	Animal wild-type #14	serum	142.84	367.52	67.08	6.75	234.5	<2.71↓	12.78	2.83	6.34	17.15	10.32	4.81	89.49	19.31
15	mouse	Animal wild-type #15	serum	94.87	698.57	38.5	3.63	251.5	<2.71↓	8.54	<0.88↓	2.39	5.55	3.59	10.95	37.37	14.45
16	mouse	Animal wild-type #16	serum	109.76	1219	17.92	<2.84↓	205.9	29.94	<2.66↓	2.23	1.48	4.16	61.08	<2.03↓	129.6	16.26
17	mouse	Animal wild-type #17	serum	118.57	1540	15.3	5.33	129.6	<2.71↓	12.06	<0.88↓	1.42	5.27	3.59	<2.03↓	115.9	<2.83↓

	Sample	IL-12(p40)	IL-12(p70)	LIF	IL-13	LIX	IL-15	IL-17	IP-10	CXCL1	MCP-1	MIP-1a	MIP-1b	M-CSF	MIP-2	CXCL9	RANTES	VEGF	TNF-a
1	Animal FSHD #1	<2.17↓	<4.71↓	<2.50↓	<7.25↓	713.3	<3.07↓	1.19	107.8	176.5	25.58	10.13	17.43	3.16	<2.79↓	108.47	5.48	<2.26↓	5.17
2	Animal FSHD #2	<2.17↓	<4.71↓	<2.50↓	562.8	631.1	<3.07↓	1.69	84.55	140.75	8.05	53.55	17.43	8.02	29.55	105.77	7.18	<2.26↓	3.38
3	Animal FSHD #3	<2.17↓	<4.71↓	<2.50↓	<7.25↓	203.7	<3.07↓	<0.88↓	90.96	83.06	4.76	<3.14↓	<4.62↓	<2.74↓	<2.79↓	108.15	5.48	<2.26↓	<3.00↓
4	Animal FSHD #4	<2.17↓	<4.71↓	<2.50↓	<7.25↓	821.4	<3.07↓	2.15	91.67	76.32	34.34	27.06	17.43	<2.74↓	<2.79↓	98.24	<4.27↓	<2.26↓	<3.00↓
5	Animal FSHD #5	<2.17↓	<4.71↓	<2.50↓	<7.25↓	1696	<3.07↓	1.54	59.62	78.88	16.24	<3.14↓	9.81	<2.74↓	<2.79↓	85.32	15.56	<2.26↓	<3.00↓
6	Animal FSHD #6	<2.17↓	<4.71↓	<2.50↓	<7.25↓	485.9	<3.07↓	<0.88↓	100.1	100.19	47.75	<3.14↓	<4.62↓	<2.74↓	<2.79↓	102.05	12.67	<2.26↓	6.6
7	Animal FSHD #7	63.96	<4.71↓	<2.50↓	20.85	1278	<3.07↓	0.99	84.72	64.3	9.71	33.47	30.09	30.49	300.59	99.07	19.21	<2.26↓	<3.00↓
8	Animal FSHD #8	2.31	<4.71↓	<2.50↓	<7.25↓	974.3	<3.07↓	1.12	94.83	124.97	16.24	10.13	<4.62↓	<2.74↓	<2.79↓	87.5	<4.27↓	<2.26↓	<3.00↓
9	Animal FSHD #9	<2.17↓	<4.71↓	<2.50↓	<7.25↓	433.6	<3.07↓	<0.88↓	70.43	20.22	<2.63↓	<3.14↓	<4.62↓	<2.74↓	12.74	76.45	<4.27↓	<2.26↓	<3.00↓
10	Animal wild-type #10	7.11	<4.71↓	<2.50↓	<7.25↓	273	<3.07↓	0.99	89.24	92.15	16.24	19.59	39.62	3.16	12.74	99.91	4.53	<2.26↓	<3.00↓
11	Animal wild-type #11	<2.17↓	<4.71↓	<2.50↓	<7.25↓	280.8	<3.07↓	<0.88↓	115.1	86.6	22.54	19.59	25.59	<2.74↓	<2.79↓	80.11	11.43	<2.26↓	<3.00↓
12	Animal wild-type #12	<2.17↓	<4.71↓	<2.50↓	<7.25↓	507.1	9.5	1.05	90.58	92.15	9.71	33.47	<4.62↓	8.02	44.55	95.2	4.53	<2.26↓	<3.00↓
13	Animal wild-type #13	<2.17↓	9.54	<2.50↓	<7.25↓	183.9	<3.07↓	<0.88↓	63.37	42.07	<2.63↓	<3.14↓	<4.62↓	<2.74↓	<2.79↓	77.43	12.98	<2.26↓	<3.00↓
14	Animal wild-type #14	210.7	72.84	<2.50↓	114	270.6	41.9	7.45	86.52	53.2	34.34	135.18	77.52	98.9	525.28	63.87	46.11	2.53	3.08
15	Animal wild-type #15	47.27	47.12	<2.50↓	52.07	2682	20.24	5.44	85.33	25.23	38.51	75.29	49.19	42.92	271.62	62.99	30.59	<2.26↓	6.6
16	Animal wild-type #16	8.53	61.94	<2.50↓	<7.25↓	2739	<3.07↓	4.18	86.44	54.42	32.92	27.06	7.11	6.13	82.84	55.2	20.19	<2.26↓	15.62
17	Animal wild-type #17	18.01	13.78	<2.50↓	43.1	1150	20.24	1.54	89.7	136.52	39.87	127.51	9.81	25.31	188.65	79.35	33.85	<2.26↓	3.68

Table S6- Cytokine/chemokine profile in the serum from 14-18 month-old chronic FSHD-like mice. Luminex protein quantification of cytokines/chemokines in the serum of 14-18 month-old chronic FSHD-like mice.
